# Chemical entity normalization for successful translational development of Alzheimer’s disease and dementia therapeutics

**DOI:** 10.1186/s13326-024-00314-1

**Published:** 2024-07-31

**Authors:** Sarah Mullin, Robert McDougal, Kei-Hoi Cheung, Halil Kilicoglu, Amanda Beck, Caroline J. Zeiss

**Affiliations:** 1 Roswell Park Comprehensive Cancer Center, Buffalo, NY USA; 2 Yale University School of Public Health, New Haven, CT USA; 3 University of Illinois Urbana-Champaign, Champaign, IL USA; 4 Albert Einstein College of Medicine, Bronx, NY USA; 5 Yale University School of Medicine, New Haven, CT USA

**Keywords:** Entity normalization, Ontology, ChEBI, Alzheimer, Dementia

## Abstract

**Background:**

Identifying chemical mentions within the Alzheimer’s and dementia literature can provide a powerful tool to further therapeutic research. Leveraging the Chemical Entities of Biological Interest (ChEBI) ontology, which is rich in hierarchical and other relationship types, for entity normalization can provide an advantage for future downstream applications. We provide a reproducible hybrid approach that combines an ontology-enhanced PubMedBERT model for disambiguation with a dictionary-based method for candidate selection.

**Results:**

There were 56,553 chemical mentions in the titles of 44,812 unique PubMed article abstracts. Based on our gold standard, our method of disambiguation improved entity normalization by 25.3 percentage points compared to using only the dictionary-based approach with fuzzy-string matching for disambiguation. For the CRAFT corpus, our method outperformed baselines (maximum 78.4%) with a 91.17% accuracy. For our Alzheimer’s and dementia cohort, we were able to add 47.1% more potential mappings between MeSH and ChEBI when compared to BioPortal.

**Conclusion:**

Use of natural language models like PubMedBERT and resources such as ChEBI and PubChem provide a beneficial way to link entity mentions to ontology terms, while further supporting downstream tasks like filtering ChEBI mentions based on roles and assertions to find beneficial therapies for Alzheimer’s and dementia.

**Supplementary Information:**

The online version contains supplementary material available at 10.1186/s13326-024-00314-1.

## Introduction

Despite advances in identifying the biological basis of Alzheimer’s disease (AD) and dementia, there are few chemical therapeutic interventions. Approved drugs are largely limited to cholinesterase inhibitors and memantine, which provide symptomatic management, and two drugs reported to reduce progression, aducanumab and lecanemab [1–3]. Animal studies tend to report high rates of success, but translation of therapies from animals to humans is generally poor. Several factors undermine the usefulness of animal studies, including insufficient rigor in animal study design, reporting and reproducibility, publication bias, over-reporting of significance, and over-reliance on non-clinical outcome measures [4–7]. The most challenging aspect of neurodegenerative diseases are their biological complexity, and the associated inability of animal models to fully recapitulate disease mechanisms [5–7]. When combined with reductionist approaches inherent in the modern scientific method, results obtained in animal models fail to translate to more complex systems (e.g. patient populations) with emergent properties [5, 8]. One approach to overcome the challenges is to assess the generalizability of therapeutic mechanisms over diverse preclinical systems. This approach is not unprecedented, and is utilized to develop therapeutics that cannot be tested in humans using the Food and Drug Administration (FDA) Animal Rule [9]. Zeiss et. al. applied this approach in the context of Parkinson’s disease by using text mining to extract translation-related characteristics across pre-clinical systems from scientific publications [10]. We build on that work by refining the methodology for capturing chemical interventions in the AD and dementia literature. This expands our capacity to associate translationally relevant data (e.g. animal model, outcome measures used to establish efficacy and biomarker data [10]) with chemical interventions to assess generalizability of interventional studies across animal systems and humans.

Extracting chemical interventions, or chemical mentions, can be done using a variety of methods. PubTator Central is an automated text mining tool that extracts entity mentions from PubMed titles, abstracts, and full text [11]. PubTator uses TaggerOne [12] to recognize chemical named entities and normalize them to Medical Subject Headings (MeSH). While MeSH is a rich vocabulary for the purpose of indexing literature, it does not contain extensive ontological assertions with linked chemical structure information [13]. In addition, a large portion of MeSH terms are supplementary chemical records (SCR) that are not organized in the tree hierarchy. Using an ontology, as opposed to a terminology like MeSH, can allow for further application of the found chemical intervention mentions. For instance, mentions can be classified based on their properties and roles or mentions can be grouped under parents using the hierarchical structure. In addition, ontologies provide the ability to link between chemical structures and their biological processes, which can be used for further downstream inference-based analysis including knowledge graph embeddings, drug design, and improved efficacy [14].

There are many additional sources of chemical information, including chemical structure databases (e.g. ChEMBL [15]), chemical literature databases (e.g. PubChem [16, 17]), and chemical ontologies (e.g. Chemical Entities of Biological Interest (ChEBI) [18]). Unlike MeSH, ChEBI is a fully curated database and OBO Foundry ontology [19] for molecular entities, containing hierarchical structure, relationships, definitions, structure information, and synonyms [18]. Therefore, using ChEBI with its associated ontological assertions and additional database information provides a marked improvement over MeSH. Normalization to ChEBI entities from PubTator Central chemical named entity mentions would be trivial if there existed a direct one-to-one map between MeSH identifiers and ChEBI identifiers. However, this is not the case. First, ChEBI identifiers tend to be more specific than MeSH identifiers. In addition, popular databases, such as DrugBank and ChEMBL, do not have MeSH identifiers directly in their downloadable databases, making it difficult to convert from one resource to another [15, 20]. Other resources, such as BioPortal and PubChem’s Identifier Exchange, allow mapping between MeSH and ChEBI [16, 21]. However, the coverage is severely lacking, only covering 14.4% of the ChEBI ontology.

Prior research concerning chemical entity normalization consists primarily of lexical or rule-based approaches [22, 23], PageRank methods [24], knowledge graph disambiguation methods [14], and vector-based methods [14]. The main source of difficulty in mining chemical mentions from the literature and normalizing them to standard identifiers is the lack of standardized naming conventions to represent the chemical structural information [14]. For example, the trivial name carvedilol has brand names (i.e., Coreg, Dilatrend), identifier numbers (CAS RN: 72956-09-3), and systematic names (e.g., $(+-)-1-(Carbazol-4-yloxy)-3-((2-(o-methoxyphenoxy)ethyl)amino)-2-propanol$, $1-(9H-carbazol-4-yloxy)-3-{[2-(2-methoxyphenoxy)ethyl]amino}propan-2-ol)$ that incorporate the structure or part of the structure. Systematic names can have variations on how hyphens, commas, or dashes are located. In addition, there can be abbreviations or acronyms and misspellings. While these methods often perform well on higher-level chemical mentions, accuracy degrades as mentions become more granular.

Here, we provide a reproducible hybrid approach that incorporates machine learning and a dictionary-based method for normalizing the chemical mentions extracted by PubTator Central. We have curated a large hierarchical synonym database from chemical databases to find candidate ChEBI entities for each textual mention. Then, we used a Bidirectional Encoder Representations from Transformers (BERT) language model-based task to identify the best entity from the candidates. Transformer models for normalization have been explored and primarily focus on non-chemical entities and normalizing to MeSH identifiers [25, 26]. For instance, PhenoTagger uses a dictionary-based method and continues training BERT models using the Human Phenotype Ontology for a classification task [27]. However, PhenoTagger requires a distantly supervised training set and generation of both positive and negative labels. Chemical mentions, as we have discussed, suffer from non-standardized naming conventions [14, 28]. This makes it difficult to automatically generate reliable true negative samples for a training set without manual curation by an expert, since an addition of a dash or number can be referencing a different chemical or an author may use a more generic term that is referencing a child chemical or a related/similar chemical. Additionally, since chemical ontologies and knowledge bases store only positive samples and have an open-world assumption, we cannot simply assume that an absence of relationship means that there is no relationship to generate negative samples. Therefore, instead of framing our disambiguation task as a classification task, we took a more unsupervised approach to incorporate the ontological structure and synonymy contained in ChEBI. In addition, we generated a context-based mapping between MeSH and ChEBI, making use of external synonym databases and ontological parent-child relationships in ChEBI, improving upon the lack of coverage found between the two resources. Finally, through this process, we identified potential new candidate entities related to AD and dementia.

## Materials and methods

### Resources used

As of December 2021, we extracted 286,484 abstracts from the approximately 30 million PubMed abstracts using the key terms ‘Alzheimer’ or ‘dementia.’

#### PubTator Central

PubTator Central uses TaggerOne for named entity recognition (NER) and normalization (entity linking) to ontologies [11, 12]. PubTator Central normalizes to MeSH for chemicals [13]. For example, Simvastatin can be mapped to the MeSH ‘Simvastatin’ with unique ID D019821. We filtered PubTator Central annotations for chemical mentions and removed mentions of ‘water.’

#### PubChem

PubChem is an open chemistry database funded by the National Institute of Health that collects chemical molecule information: chemical structures, identifiers, chemical and physical properties, and synonyms [16]. We extracted synonyms, listings of the names aggregated from all substances whose standardized form is the compound identifier (CID). We removed names that have inconsistent structure.

#### Chemical Entities of Biological Interest (ChEBI)

ChEBI (http://www.ebi.ac.uk/chebi) is available as a database and an ontology for molecular entities with a focus on small chemical compounds that are products in nature or synthetic products used to intervene in the processes of living organisms [18]. ChEBI is part of the OBO Foundry with Basic Formal Ontology as the upper level ontology, meaning that the ontology is well-formed and interoperable with other OBO Foundry ontologies such as the Gene Ontology (GO; http://geneontology.org) and Protein Ontology (PRO; https://proconsortium.org), allowing for linkage between chemicals and biological processes. ChEBI release 201 has 143,263 entities with 59,214 fully annotated (placement within the hierarchical ChEBI structure and definitions). In addition, it has almost 300,000 relationships. For example, Simvastatin can be mapped to CHEBI:9150. ChEBI provides relational information such as Simvastatin ‘$is\ a$’ ‘CHEBI:40303 lovastatin’ and ‘$has\ role$’ ‘CHEBI:50266: prodrug’. Status ‘C’ (curated by a ChEBI team member and released in the public version) or ‘E’ (exists, but has not been curated by a ChEBI team member) were retained. The statuses removed were entities just submitted, deleted, or obsolete. Once these statuses were filtered, we retained either a 2 or 3 star rating, with a 3-star rating having been annotated manually by the ChEBI team and a 2-star rating having been manually annotated by a third party [18]. We incorporated entities not fully curated in order to capture the breadth of possible entities in the PubMed literature. This resulted in 112,658 unique entities.

### Data pre-processing and hierarchical dictionary method

We then matched the set of textual mentions found by the chemical TaggerOne model in PubTator Central to a set of candidate ChEBI entities. Figure 2 illustrates the schema pipeline for matching the set of textual mentions found by to a set of candidate ChEBI entities and subsequently disambiguating them. Similar to the tools tmChem and NLM-CHEM used by PubChem, we take the hierarchical dictionary method approach [22, 23]. Pre-processing was done in a hierarchical manner such that exact matches were used first. Then, we performed data cleaning: lemmatization, lower-case, abbreviation resolution and removal of dashes, parentheses and commas. We used the Schwartz-Hearst algorithm for identifying abbreviations (Python package abbreviations) in each abstract. Abbreviations were then replaced before the candidate list was selected. Since our task deals with chemical entity mentions, we did not stem because this may remove an important part of the chemical meaning. Finally, if no match occurred, we implemented fuzzy-string matching. We have used the following hierarchical method such that once a result is obtained within a level of the hierarchy, the search for candidate ChEBI entities stops. The hierarchy follows (1) exact match to ChEBI name or synonym (2) relaxed match to ChEBI name or synonym which was retained as possible candidate for disambiguation (3) any match obtained by exact or relax match to PubChem or a fuzzy- string match to PubChem or ChEBI was used as potential candidate entities and disambiguation was done.

#### Exact match to ChEBI name

First, we extracted the ChEBI name from the ChEBI ontology. This is the name recommended for use by the biological community and conforms to current International Union of Pure and Applied Chemistry (IUPAC) recommendations of chemical nomenclature [18]. For example, the title ‘Investigation of Low Dose Cabazitaxel Potential as Microtubule Stabilizer in Experimental Model of Alzheimer’s Disease: Restoring Neuronal Cytoskeleton’ extracted the named entity ‘Cabazitaxel,’ which was mapped to ChEBI:63584.

#### Exact match to ChEBI synonym

Then, we extracted the ChEBI synonym list which consists of alternative names for an entity derived from external sources or devised by the annotators based on recommendations of IUPAC, Nomenclature Committee of the International Union of Biochemistry and Molecular Biology (NCIUBMB), or their associated bodies. For example, the title ‘Novel analogues of chlormethiazole are neuroprotective in four cellular models of neurodegeneration by a mechanism with variable dependence on GABA(A) receptor potentiation’ extracts the named entity ‘chlormethiazole,’ which is linked to a candidate entity ChEBI:92875 with ChEBI name ‘5-(2-chloroethyl)-4-methylthiazole.’

#### Relaxed match

Using Python 3.8.10 and the Natural Language Toolkit(NLTK) package we removed punctuation, lower-cased the text, and lemmatized, removing pluralization. For example, the pluralized entity mention ‘$\alpha$-keto esters’ becomes ‘$\alpha$-keto ester’ and is matched to CHEBI:51848 ‘$\alpha$-ketoester’ from the title ‘Oxidative cross-dehydrogenative [2 + 3] annulation of $\alpha$-amino ketones with $\alpha$-keto esters: concise synthesis of clausenamide analogues.’

#### PubChem and other resources synonym dictionary

Synonyms in PubChem can be attributed to multiple PubChem compound identifiers (CIDs) and therefore, with the remaining unmatched entity mentions, we did an exact match, followed by a relaxed match, to the PubChem filtered synonym list for the ChEBI entity’s associated CID. This procedure often produced multiple possible ChEBI matches for which entity disambiguation was done as described in the next section. For example, the title ‘The therapeutic effect of kavain and magnesium orotate on traumatic and vascular brain lesions’ had the named entity ‘kavain’ extracted. Using the set of synonyms provided by PubChem, this maps to ChEBI:6117 ‘kawain,’ ChEBI:91863 ‘4-methoxy-2-(2-phenylethenyl)-2, 3-dihydropyran-6-one’, and ChEBI:92164 ‘(2R)-4-methoxy-2-(2-phenylethenyl)-2, 3-dihydropyran-6-one.’

Finally, on the remaining entity mentions we leveraged the Python package fuzzywuzzy using the Levenshtein Distance (an edit distance) and kept candidates with a ratio greater than $50\%$, matching in the same order of the hierarchical process: ChEBI name, ChEBI synonym, PubChem synonym. Fuzzy matching can produce an intractable and inefficiently large number of candidates. Therefore, we have included options to set the ratio larger than $50\%$ and to set a threshold for the maximum number of candidates to include per mention ranked by Levenshtein Distance. For the entity mentions relying on fuzzy matching, the models were run with 500 top-ranked candidates by Levenstein distance for each mention to minimize computational time and scalability. Given the degradation after 500 matches (Supplement 1) and since it takes 0.136 seconds for each additional candidate using CPUs and 0.0267 when GPUs are enabled, we chose to keep 500 matches to balance efficiency, accuracy, and completeness.

### Entity disambiguation for generated candidates by dictionary method

**Fig. 1:**
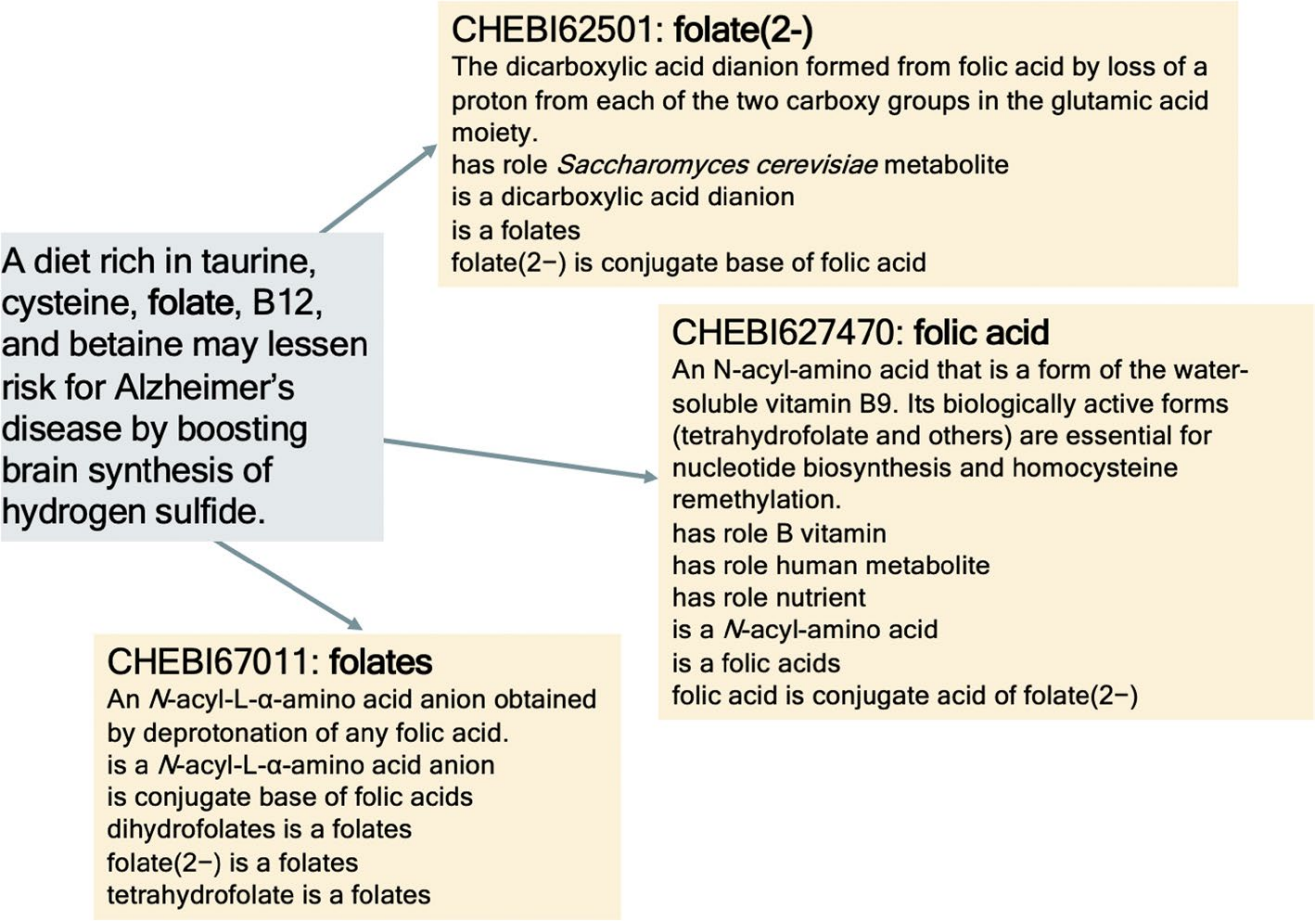
Folate Entity Disambiguation. A mention of folate (blue box) can be mapped to multiple ChEBI terms (yellow boxes). The mention refers to the supplement, meaning that the mention should be disambiguated to ‘CHEBI:627470: folic acid’

**Fig. 2:**
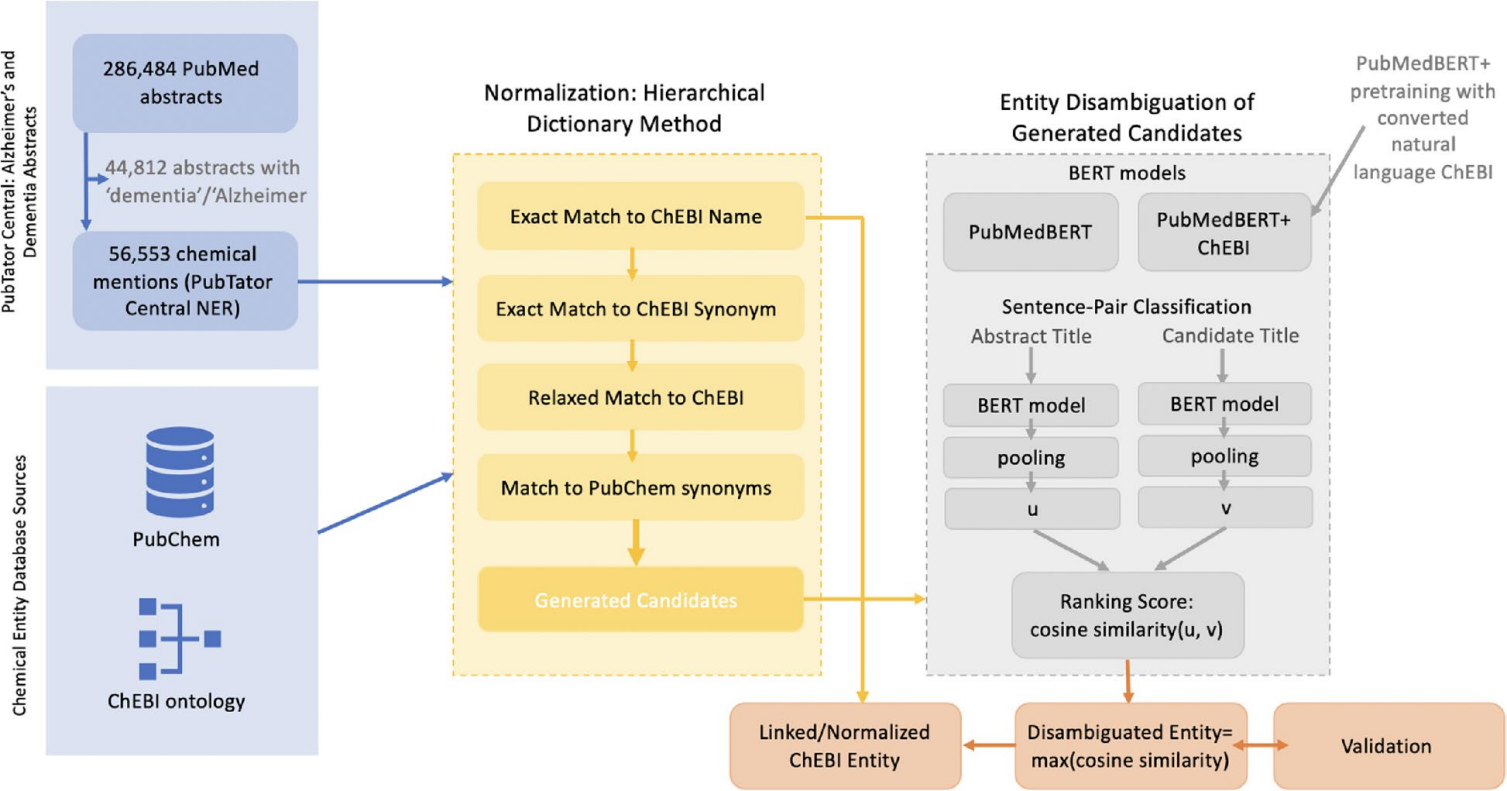
ChEBI entity normalization pipeline. 286,484 PubMed Abstracts were queried with the keywords ‘Alzheimer’ and ‘Dementia’ resulting in 56,553 chemical mentions. Using chemical entity database resources (ChEBI ontology, PubChem), a hierarchical dictionary-based method was used to generate ChEBI entity candidates. These candidates were disambiguated using a sentence-pair classification task where they were ranked by cosine similarity. We developed two models for this (1) using the pretrained PubMedBERT and (2) continuing pretraining on PubMedBERT using ChEBI converted into natural language. The maximum cosine score between the original named entity and the candidate was retained. Our method was validated using our annotated gold standard dataset and compared to the MeSH normalized TaggerOne mentions

Figure 2 depicts the pipeline for normalization to ChEBI entities. Using our hierarchical dictionary and processing method, we disambiguated between a set of candidate ChEBI entities. If there was an exact match between the entity mentions and any entity, we retained that entity and no candidate list was built. However, for relaxed matches, matches using PubChem, and matches using fuzzy string matching, a set of candidates was produced. For example, folate can refer to either folate in the body, namely serum folate, or folate taken as a supplement, folic acid, providing us with two candidate entities. In Fig. 1, ‘a diet rich in taurine, cysteine, folate, B12, and betaine may lessen risk for Alzheimer’s disease by boosting brain synthesis of hydrogen sulfide’ refers to the supplement, meaning that the mention should be disambiguated to ’CHEBI:627470: folic acid’.

We formulated entity disambiguation of the generated candidates as a sentence-pair classification task using contextual information in the title and information from ChEBI’s ontological structure. A visual interpretation of this can be found in the gray box in Fig. 2. Transformer architectures such as BERT, pretrained on large amounts of text in an unsupervised manner, have advanced the state-of-the-art in many NLP tasks, including entity normalization [25, 29]. We used PubMedBERT, a BERT variant trained from scratch on PubMed abstracts and PubMed Central full-text articles, shown to outperform baseline BERT, as our base model to do additional pretraining with the ChEBI ontology converted to natural language form [30–32]. Therefore, the pipeline follows: (1) pretraining BERT model for entities using the ChEBI ontology, (2) train a sentence-level BERT model, (3) calculate cosine similarity for each candidate.

To continue pretraining PubMedBERT with the ChEBI ontology (1), we converted ChEBI into natural language. For each triple, synonym, and definition contained in the ChEBI ontology for an entity, we created a human-readable sentence (e.g. The set of triples: diacylglycerol 44:4 | $is\ a$ | diglyceride and diacylglycerol 44:4 | SYNONYM | DAG 44:4, becomes the human readable natural language sentence ‘diacylglycerol 44:4, otherwise known as DAG 44:4, is a diglyceride’). Relations and synonyms were given natural language equivalents, such that ‘has_part’ became ‘is partially made up of’ and ‘IUPAC NAME’ was transformed into ‘has preferred name.’ These natural language chunks for each entity were then used as a validation and training set to continue pretraining PubMedBERT with a sample size of 164,849 human readable sentences. We used the huggingface transformers 4.14.0 package, the initial ‘microsoft/BiomedNLP-PubMedBERT-base-uncased-abstract-fulltext’ PubMedBERT model, and PyTorch 1.10.0 with an initiated learning rate of 0.0001 on 3 epochs with a batch size of 8 and a final train loss of 0.149 and 0.203 on the validation set. All other hyperparameters were set to the default fixed values.

In Fig. 2, we show the sentence-pair classification task, for entity disambiguation. The original abstract title was compared with the title replacing the original term with the candidate entity name (candidate title). Cosine similarity was then calculated for each candidate using a sentence embedding algorithm, Sentence-BERT, and the top candidate selected [33]. To get a sentence embedding to compare our candidate entities, we trained Sentence-BERT on our PubMedBERT embedding that was pretrained on the ChEBI ontology using the Semantic Textural Similarity (STS) task (PubMedBERT+ChEBI) [33]. The STS task assigns a score based on the similarity of two sentences, using the STS benchmark dataset, which is split into a  67/17/16 train/validation/test split, and sentence-transformers 2.1.0 [34, 34]. The model was trained for 4 epochs with a training batch size of 16. Like PubMedBERT, the max number of tokens was 512. Mean pooling was applied to get the sentence vectors. The STS validation set had a Pearson correlation of 0.8315 and a Spearman correlation of 0.832 for cosine similarity. On the STS testing set, the model had a Pearson correlation of 0.796 and a Spearman correlation of 0.79 for cosine similarity.

In comparison to the method PubMedBERT+ChEBI, the method ‘PubMedBERT’ refers to the PubMedBERT model trained using the Sentence-BERT methodology on the STS dataset.

### Validation and analysis

To create a gold standard for comparison, we randomly selected 500 titles from our 286,484 PubMed abstract entries. A domain expert (CJZ) labeled if the chemical intervention entity mentions identified by TaggerOne were accurate from the abstract titles. Then, a PhD in Biomedical Informatics (SM) linked these extracted entity mentions with ChEBI entities. We report overall accuracy of attaining the correct ChEBI entity when compared to the gold standard (n=484). In addition, we report precision, recall, and the F1 score for our method when compared to the manually curated gold standard with the outcome of whether or not an entity was present in the ChEBI ontology for the extracted mention with 3.2% of entities not having a ChEBI entity match. This allows us to decipher whether or not we can create a model, dependent on a threshold, that is able to find if there even exists a ChEBI entity for the specified chemical mention. To assess the sensitivity of choosing a cosine similarity threshold as a way to flag potentially incorrect matches where a true ChEBI entity either does not exist or was not in the generated candidate set, we analyze each accuracy measure at different cosine similarity thresholds. In addition, since PubTator Central is already normalized to MeSH terms, we analyzed the mapping and differences in coverage from the MeSH terms extracted by PubTator Central to our normalized ChEBI entities using BioPortal [16, 21].

Finally, to show the applicability and utility of the method outside of this use case, we analyzed our methodology on the “Colorado Richly Annotated Full-Text” (CRAFT) corpus [35], a set of 67 full-text biomedical articles from PubMed Central Open Access subset, that contains 4,548 manual annotations of ChEBI entities. In this work, we used version 3.0 of this corpus and changed it slightly to fit the scope of our algorithm: we removed mentions that were 2 characters or less, annotated ChEBI entities that fell outside of status’ C and E and stars 2 and 3, and terms that pertained to genes or proteins or were too generic (ie., amyloid, messenger, message, water, aqueous, feed, chow) for a total of 4,066 annotations. The processing of the corpus can be found on GitHub. For contextual disambiguation, we extracted the sentence that contains the mention.

### Baseline normalization methods

For comparison, we have selected publicly available normalization methods to ChEBI: Relation Extraction for Entity Linking (REEL) [24] and Gilda [36] for assessment on our gold standard and CRAFT. Other normalization methods exist, but code was not publicly accessible [37, 38]. REEL is based on the personalized PageRank (PPR) algorithm and builds a disambiguation graph with nodes as candidates for the entities and added edges according to the relations in the text. The PPR algorithm and the information content of ChEBI are then applied to choose the candidate for each entity that maximises the coherence of the disambiguation graph. The code is publicly available at: https://github.com/lasigeBioTM/REEL. Gilda first implements a grounding algorithm inspired by [39] that allows for efficient approximate matches to any of the terms appearing in the resource table, which includes ChEBI, and then uses logistic regression classification models and Adeft [40] for context aware disambiguation. For the Alzheimer and dementia gold standard, we used titles for disambiguation and for CRAFT we used the sentence that contains the mention. Gilda is available at: https://github.com/indralab/gilda.

## Results

From 286,484 PubMed abstracts, we extracted 44,812 unique abstracts identified by their PubMed identifiers (PMIDs) that contained chemical entity mentions. These 44,812 abstracts titles had a mean of 1.279 chemical entity mentions per title (sd=0.591) and a total of 56,553 chemical mentions.

### Hierarchical dictionary method

The results of the hierarchical dictionary method can be found in (Table 1). Twenty-eight thousand eight hundred eighty-one mentions matched one ChEBI name exactly and 9,335 matched a ChEBI synonym exactly. Another 3% matched after relaxing lemmatization and punctuation. One hundred ninety-four entities were flagged as not matchable due to the extracted entities having two or less characters.

**Table 1:** Hierarchical dictionary method results: single ChEBI entity selected

	n(%)
Exact:ChEBI Name	28,881(50.9)
Exact:ChEBI Synonym	9,335(16.5)
Relaxed:ChEBI Name and Synonym	1,736(3)
Exact:PubChem	1,980(3.5)
Relaxed:PubChem	226(0.4)

### Candidate selection and disambiguation

Sixteen thousand nine hundred fifty-five entities needed disambiguation with a median of 4 (IQR=8) candidates per entity. This produced 99,378 total sentence-pairs of title and title replaced with candidate entity.

Using our method of disambiguation, pretraining PubMedBERT on the ChEBI ontology and subsequently training a Sentence-BERT model on the Semantic Textual Similarity (STS) task (PubMedBERT+ChEBI), we retained the maximum cosine similarity score between the sentence-pairs. The final retained candidate entities had a cosine similarity median of 0.97 (IQR=0.051). For example, TaggerOne identified the mention ‘epicatechin’ in the paper titled ‘Dietary (-)-epicatechin as a potent inhibitor of $\beta \gamma$-secretase amyloid precursor protein processing’ [41]. First, finding no exact match to a ChEBI entity, our method created a candidate entity list: ‘CHEBI:15600 (+)-catechin’,‘CHEBI:90 (-)-epicatechin’, ‘CHEBI:76125 (+)-epicatechin’, and ‘CHEBI:23053 catechin.’ Next sentence pairs were created for each of these entities such that the first sentence was the title and the paired sentence replaced the mention ‘epicatechin’ with each of the entity names. This resulted in Cosine similarities: 0.918, 0.985, 0.984, and 0.927, respectively for PubMedBERT+ChEBI. The maximum cosine similarity normalizes the mention to ‘CHEBI:90 (-)-epicatechin’, which is the correct mention based on the context surrounding the mention in the title. For PubMedBERT without pretraining on the ChEBI ontology, the resulting cosine similarities are 0.997, 0.998, 0.998, and 0.999, respectively, choosing the less specific parent term ‘CHEBI:23053 catechin.’

Mentions that had a low cosine similarity tended to have a ChEBI exact name that was systematic, incorporating the structure in the name or were abbreviations. For instance, the extracted named entity ‘Suloctidil’ was normalized to the correct candidate ChEBI entity ‘CHEBI:91639 2-(octylamino)-1-[4-(propan-2-ylthio)phenyl]-1-propanol’ and had a cosine similarity of 0.531. Not surprisingly, a larger portion, 2.15% of entities were normalized to ‘CHEBI:53289 donepezil’ and 1.43% of mentions were normalized to ‘CHEBI:64312 memantine’, approved drugs for AD. Additionally, lipids and cholesterol, including fatty acids, as well as herbal supplements like curcumin and melatonin were highly mentioned, which corresponds to the known AD literature [42].

### Comparison to the gold standard and CRAFT

**Fig. 3:**
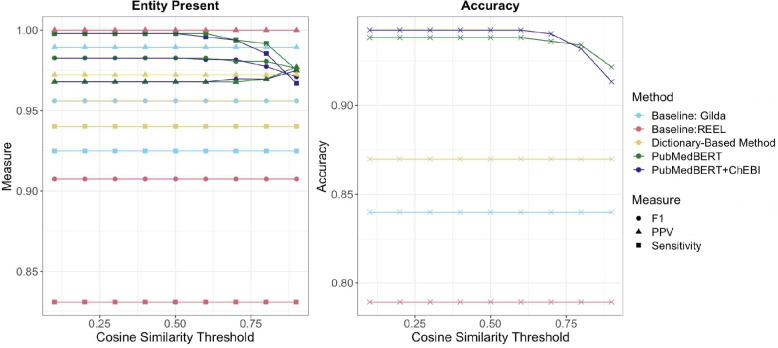
Gold Standard Comparison to Hierarchical Dictionary and Disambiguation Methods. Measures of Accuracy for whether or not an entity is present in the ChEBI ontology and a match can be made (left panel) show highest positive predictive value (PPV) and F1 for the lowest thresholds for both BERT-based methods. The baseline models, REEL (PPV=1, F1=0.907), Gilda (PPV=0.989, F1=0.956), and dictionary-based method alone (PPV=0.972, F1=0.956), were more precise than the BERT-based models (PPV=0.967). Overall accuracy (n=484, 18% disambiguated entities, right panel) was maximum 0.942 for PubMedBERT+ChEBI, 0.938 for PubMedBERT, and 0.87 for the dictionary-based method alone. Disambiguated accuracy was highest for PubMedBERT+ChEBI with a maximum of 0.724 with a difference of 25.3 percentage points between PubMedBERT+ChEBI and the dictionary-based method alone. Additionally, PubMedBERT+ChEBI and PubMedBERT outperformed both baseline models with an accuracy of 0.869 for Gilda and 0.789 for REEL

To compare the randomly sampled gold standard titles and annotated ChEBI entities, we looked at three disambiguation methods: fuzzy string matching, PubMedBERT, and PubMedBERT+ChEBI. Of the 484 randomly selected and annotated entities, 87 needed to be disambiguated after dictionary matching. For the dictionary-based method, we disambiguated the terms that had more than one potential candidate using fuzzy matching [43]. We kept the largest ratio of the Levenshtein Distance fuzzy match ratio. This is held constant across all cosine similarity thresholds. The final selected entities had a median of 75% (IQR=33). Whether or not an entity was present in the ChEBI ontology remained relatively the same across all disambiguation methods (Fig. 3). As expected, the dictionary-based method with fuzzy matching disambiguation had a lower recall (0.94) compared to the BERT language model methods (PubMedBERT:0.998, PubMedBERT+ChEBI:0.998) when a corresponding entity exists in the ChEBI ontology. PubMedBERT+ChEBI and PubMedBERT were also precise (Precision:0.97), showing that these model are able to distinguish between whether or not a mention corresponding to an entity exists in the ChEBI ontology with improved recall.

Our approach of using a Sentence-BERT model and pretraining PubMedBERT with ChEBI improves disambiguation accuracy of the correct ChEBI entity with a difference of 25.3 percentage points between PubMedBERT+ChEBI and fuzzy string matching with no threshold and an improvement of 2.3 percentage points between PubMedBERT+ChEBI and PubMedBERT (Fig. 3). In comparison to our baseline methods REEL and Gilda, our models were less precise (Fig. 3), but they had the highest sensitivity. For accuracy of finding and assigning normalized entities, PubMedBERT+ChEBI and PubMedBERT greatly outperformed both baseline models with an overall maximum accuracy of 0.942 for PubMedBERT+ChEBI and 0.936 for PubMedBERT compared to 0.869 for Gilda and 0.789 for REEL.

Figure 3 depicts that not constraining the disambiguation method with a cosine similarity threshold has the highest overall and disambiguation accuracy. This could be influenced by the minimum cosine similarity values within the gold standard set: the minimum cosine similarity found after normalization was 0.659 for PubMedBERT and 0.564 for PubMedBERT+ChEBI.

For the CRAFT annotated corpus, PubMedBERT and PubMedBERT+ChEBI maintained improved accuracy (91% and 91.17%) for normalizing mentions to ChEBI entities compared to Gilda (78.4%) and REEL (76.29%) (Table 2). Our dictionary-based method alone performed well also (88.6%) in comparison to Gilda and REEL, indicating that compiling ChEBI with other large knowledge sources like PubChem for normalization is highly beneficial. Out of 4,066 mentions in CRAFT, 551 mentions needed to be disambiguated. The final cosine similarity for these mentions was very high (PubMedBERT+ChEBI: median=0.992 (IQR:0.023), PubMedBERT: median=0.989 (IQR:0.027)), potentially indicating the discriminating power between candidates was good. The main entries (*n*=74) that our algorithm did not correctly normalize were mentions of ‘molecule’ and ‘molecules’. CRAFT annotated these as ‘CHEBI:36357 polyatomic entity’ and our algorithms identified this as ‘CHEBI:25367 molecule.’ The second highest were mentions of ‘cocktail’ (*n*=20), which incorrectly normalized to ‘CHEBI:27958 cocaine.’ Since this algorithm is dependent on similarity, it is not surprising that the other meaning of cocktail, pertaining to an alcoholic beverage, had high similarity to the illicit drug cocaine.

**Table 2:** Accuracy of normalization methods for the Alzheimer/dementia gold standard and CRAFT

Method	Gold Standard	CRAFT
Baseline: Gilda	0.869	0.784
Baseline: REEL	0.789	0.763
Dictionary-Based Method	0.87	0.886
PubMedBERT	0.942	0.91
PubMedBERT+CHEBI	0.936	0.912

## Discussion

### Candidate unlinked terms

After our hierarchical process was complete, $4.16\%$ of named entity mentions recognized by PubTator Central remained unlinked. This included specific Amyloid-beta (A$\beta$) animal models as opposed to drugs and incorrectly extracted mentions, such as ‘q & a’, ‘biomedicine’ and ‘Pytorch’. In addition, this led to multiple terms that could potentially be added to ChEBI. These primarily were newly published drugs, with literature published in the last three years, or experimental drugs. These candidate mentions are included in Table 3. In addition, some of the experimental drugs had identifiers in the chemical supplement to MeSH or were stubs or not fully annotated in PubChem or DrugBank. However, others, denoted by the stars in the table, were not contained in any previously mentioned chemical databases. Therefore, this methodology can simultaneously help curate candidate ChEBI terms and synonyms for addition to the ontology and knowledge base. If the goal is to link all possible mentions, methods for suggesting approximate or similar ChEBI entities, in terms of the hierarchy and relations contained in ChEBI or other external knowledge bases, could be adapted [44].

**Table 3:** Candidate entities that can potentially be added to ChEBI concerning dementia and Alzheimer’s chemical interventions. ^a^denotes mentions not contained in any previously mentioned chemical databases

triheptanoin	organosiloxanes^a^
bapineuzumab	semagacestat
solanezumab	tiapride
4-n-phenyl aminoquinoline^a^	tianeptine
benzoquinolizidine^a^	tramiprosate
remacemide	suloctidil
davunetide	remoxipride
rilapladib	ramelteon
cerebrolysin	praziquantel
chf5074	praxilene
idalopirdine	naftidrofuryl
fluspirilene	neramexane

### Comparison to BioPortal mapping

BioPortal contains 14,450 mappings between MeSH and ChEBI. BioPortal mappings can provide multiple ChEBI entities per MeSH term with 350 of the mentions containing multiple mappings. For example, the paper titled ‘Novel sulfamate derivatives of menthol: Synthesis, characterization, and cholinesterases and carbonic anhydrase enzymes inhibition properties,’ PubTator normalized the entity mention ‘menthol’ to MeSH:D008610 which maps to ‘CHEBI:15409 (-)-menthol’, ‘CHEBI:76306 (+)-menthol’, and ‘CHEBI:76310 (±)-menthol’, depending on the contextual information surrounding the mention [45]. However, the title and the abstract do not refer to any of these specific entities, and therefore, our model instead maps to the less specific parent entity ‘CHEBI:25187 p-mentan-3-ol’ (Cosine Similarity: 0.941). Interestingly, the model infers that menthol and p-menthan-3-ol are synonyms and that a more specific entity, such as those provided by BioPortal’s mapping, based on the context of the title cannot be justified.

After removing duplicate mappings (mappings that were one to many) by prioritizing matched entity mappings, $42.5\%$ of entities found from the mapping and our normalization matched. PubTator Central and our method did not find normalized entities for $3.88\%$ of the mentions. Entities that did not match improved from our use of disambiguation and sentence embeddings. These tended to match a more specific entity compared to BioPortal’s less specific entity. For instance, the mention ‘24S-hydroxycholesterol’, was mapped from MESH:C044563 to ‘CHEBI:50515 24-hydroxycholesterol’ by BioPortal, as opposed to the more specific term found by our method ‘CHEBI:34310 (24S)-24-hydroxycholesterol.’ BioPortal was able to map from the MeSH terms $0.67\%$ additional mappings that we normalized incorrectly with these primarily being brand names such as Aricept and acronyms that were missing from our synonym database (e.g. THA is an acronym for tacrine). Finally, we were able to find potential mappings between MeSH and ChEBI for $47.07\%$ of the mentions not in the BioPortal mappings, including investigational drug ‘Ladostigil’ (MESH:C423264 to CHEBI:177484) which is linked to studies on mild cognitive impairment and 25-hydroxy Vitamin D (MESH:C104450 to CHEBI:17933). These mappings are available on GitHub: https://github.com/sarahmul/CHEBINormalizer.

Finally, mapping directly from the normalized MeSH term to the ChEBI entity can produce incorrect entity normalization if there does not exist a more specific MeSH term. Here, ‘sulfamate’ maps to ‘MESH:C005741 sulfamic acid.’ The term ‘sulfamate’ exists as an entry term, but not as its own entity. In addition, since this is a Supplementary Concept Records (SCR), this MeSH entity does not exist in the hierarchy, and therefore, it cannot be mapped to a parent entity. Therefore, using the BioPortal mapping, we get the incorrect normalized entity, ‘CHEBI:9330 sulfamic acid.’ Our model, directly mapping from the mention, maps to the ChEBI term ‘CHEBI:131822 sulfamate.’

### Use of entity normalization in synthesizing chemical mentions for AD and dementia

Being able to classify chemical mentions in literature into possible therapeutic interventions or other important roles pertaining to AD and dementia is key for future prospective research. When a ChEBI role relation was found, we mapped the normalized ChEBI entity to parent role terms (e.g. ‘CHEBI:52217 pharmaceutical’, ‘CHEBI:33284 nutrient’). 40.87% of the mentions (out of *N*=55,765 matched mentions) can be classified as metabolites, 34.91% can be classified as pharmaceutical drugs, and 10.83% can be classified nutrients.

**Fig. 4:**
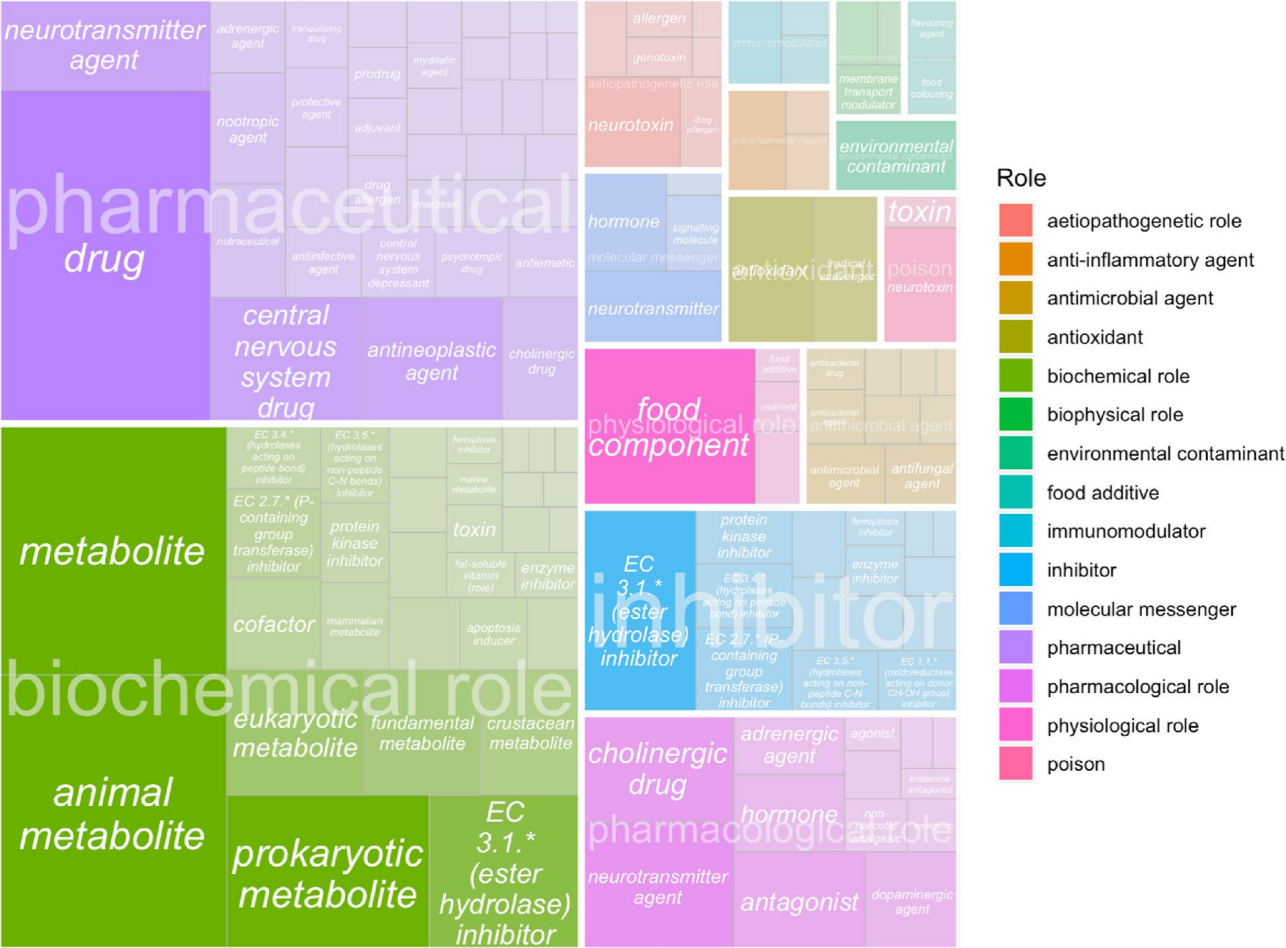
Treemap of ChEBI roles for mapped entities. A treemap with overall ChEBI parent role terms such as pharmaceutical and inhibitor (shown in the legend) overlayed on top of the children roles are presented in this figure. The darker the shade, the higher the number of times these roles occurred in our database

Figure 4 shows a treemap with overall ChEBI parent role terms such as pharmaceutical and inhibitor overlayed on top of the childrens’ roles. The darker the shade, the higher the number of times these roles occurred in our database. 99.3% of our mentions had a biochemical role including metabolites and 79.44% were pharmaceuticals including diagnostic purposes or drugs. Other primary roles included inhibitors,food components, or supplements. Pharmacological roles that had high case counts are neurotransmitter agents with 3,329 chemical entities linked to this role, cholinergic drugs, adrenergic agents, dopaminergic agents, and hormones. We were then able to filter out specific categories that were not chemical interventions using this strategy, including diagnostic chemicals such as ‘Iofetamine’.

### Limitations

This work focuses on normalization of entities once NER is done by TaggerOne, which reports a F1 score of 0.914 and normalization F1 score 0.895 on the chemical corpus, BioCreative 5 CDR [12] and entities captured were sometimes only partial entities (e.g. ‘Galantamine hydrobromide’ should have been extracted as the named entity, but only ‘Galantamine’ was extracted). Future work could account for joint NER and normalization or looking into other methods of NER before utilizing BERT models for disambiguation.

PubMedBERT was initially trained on PubMed abstracts and full-text articles from PubMed Central. While the model vocabulary contains most biomedical terms and subterms, especially those found in PubMed texts, it may not contain some of the synonyms and vocabulary terms found in ChEBI and PubChem. This could impact pretraining PubMedBERT on ChEBI. Future work should look at how to incorporate knowledge base vocabulary and synonyms, such as is contained in ontologies like ChEBI, in language models.

In addition, further work should be done creating a training corpus using techniques to convert an ontology into natural language generation [46]. This avenue of research requires additional rigorous training and validation. Creation of a ChEBI ontology natural language corpus could be advantageous.

Finally, the improved accuracy afforded by PubMedBERT+ChEBI and PubMedBERT comes at a cost in terms of computational time. To parse the gold standard data set, maximizing 500 candidates per mention, took 4.5 hours on 8 CPU cores with 16 GB of memory (mirroring a typical personal computer) and 13 minutes enabling a GPU.

## Conclusion

Use of language models, especially a model pretrained on the ChEBI ontology, combined with a dictionary-based method can provide a beneficial way to disambiguate entities. This method can be used with other ontologies or across domains that have a similar semantic structure to chemicals, such as genes and proteins.

While MeSH is a rich vocabulary, it does not contain extensive ontological assertions with linked chemical structure information and therefore, being able to normalize to ChEBI can provide useful applications. We demonstrated the usefulness of ChEBI assertions and roles for filtering AD and dementia interventions and the ability to use this ontology for effective entity normalization. Our entity normalization method found additional mappings between MeSH and ChEBI, based on contextual information. Finally, through textual data mining, we have found additional candidate terms that can be added to ChEBI.

### Supplementary Information

Supplementary Material 1.

## Data Availability

The datasets and code generated and/or analysed during the current study are available at https://zenodo.org/badge/latestdoi/596715128 and https://github.com/sarahmul/CHEBINormalizer upon publication.

## Abbreviations

ChEBI: Chemical Entities of Biological Interest
AD: Alzheimer’s disease
FDA: Food and Drug Administration
MeSH: Medical Subject Headings
SCR: Supplementary chemical records
BERT: Bidirectional Encoder Representations from Transformer
NER: Named entity recognition
CID: Compound identifier
IUPAC: International Union of Pure and Applied Chemistry
NCIUBMB: Nomenclature Committee of the International Union of Biochemistry and Molecular Biology
NLTK: Natural Language Toolkit
A$$\beta$$: Amyloid-beta
CRAFT: Colorado Richly Annotated Full-Text
