# Supplementary material for: Chemical entity normalization for successful translational development of Alzheimer’s disease and dementia therapeutics
Source: J Biomed Semantics. 2024 Jul 31;15:13. doi: 10.1186/s13326-024-00314-1 (PMC11290083; doi:10.1186/s13326-024-00314-1)
Supplement: Supplementary file 1 — Supplementary Material 1. [file 13326_2024_314_MOESM1_ESM.docx]

Supplement 1: Experiments for choosing the number of candidates

Fuzzy matching can produce an intractable and inefficiently large number of candidates. Therefore, we have included options to set the ratio larger than 50 and to set a threshold for the maximum number of candidates to include per mention ranked by Levenshtein Distance. We set a candidate set of 500 due to computational time.

There were 8,724 mentions in our total Alzheimer dataset minus the gold standard (n=25) that used fuzzy matching (meaning, no candidate was found through the other hierarchical matches). Below is a histogram of the total number of candidates produced from fuzzy matching with a median of 383.5 candidates (IQR=1308.25).


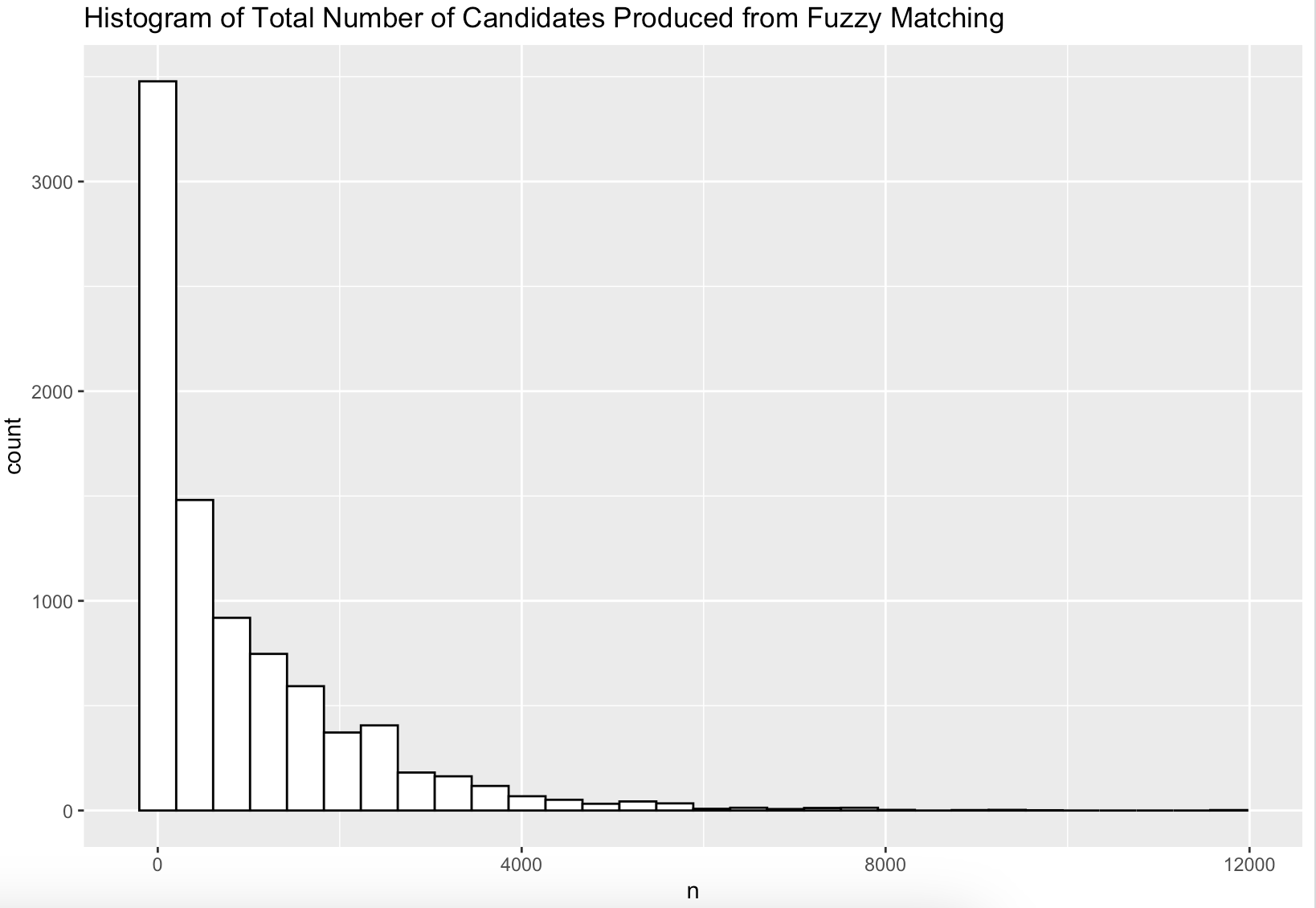


Supplement Figure 1. Histogram of the total number of candidates produced by fuzzy matching in the Alzheimer’s and Dementia Dataset minus the gold sample mentions

Embedding the sentence containing the candidate and the sentence containing the mention and then calculating cosine similarity led to computation time of approximately 0.136 seconds with only CPUs for each additional candidate added and on average 0.0267 when GPUs are enabled for part of the computation (Supplement Table 1). Additionally, we have provided a way to set your own threshold, if this computational complexity is not feasible, especially given the diminishing returns shown below.

| Threshold Set | Number of candidates | Time (seconds) |
| --- | --- | --- |
| 100 | 719717 | 18683.8533 |
| 200 | 1287258 | 33434.2878 |
| 300 | 1780980 | 46146.9309 |
| 400 | 2229081 | 57516.0702 |
| 500 | 2646857 | 71359.2647 |

Supplement Table 1. Experiment for setting a candidate number threshold for fuzzy matching based on time in seconds it takes the candidates to run through a GPU-enabled pipeline.

Additionally, the more candidates allowed by fuzzy match, the higher departure from an actual match, especially for mentions that are short. Below is an example of PMID: '33667517' and the mention:'fat cholesterol’, which has 1,149 entries. There is an extremely high chance that in this example, one of the top 10 choices is the correct ChEBI entity. Looking past index 500, we see that the ‘possible’ column and the ‘ChEBI name’ column are not associated with the mention. Going past 500 has diminishing returns.

Supplement Table 2. Example of fuzzy match degradation as the number of matches increases.

| **Ind** | **pmid** | **term** | **ChEBI** | **score** | **possible** | **ChEBI name** |
| --- | --- | --- | --- | --- | --- | --- |
| 1 | 33667517 | fat cholesterol | 16113 | 85 | cholesterol | cholesterol |
| 2 | 33667517 | fat cholesterol | 47774 | 80 | ldl cholesterol | low-density lipoprotein cholesterol |
| 3 | 33667517 | fat cholesterol | 47775 | 80 | hdl cholesterol | high-density lipoprotein cholesterol |
| 4 | 33667517 | fat cholesterol | 79839 | 80 | allocholesterol | 3beta-Hydroxycholest-4-ene |
| 5 | 33667517 | fat cholesterol | 16608 | 77 | cholestenol | 5alpha-cholest-8-en-3beta-ol |
| 6 | 33667517 | fat cholesterol | 17168 | 77 | lathosterol | 5alpha-cholest-7-en-3beta-ol |
| 7 | 33667517 | fat cholesterol | 47773 | 77 | vldl cholesterol | very-low-density lipoprotein cholesterol |
| 8 | 33667517 | fat cholesterol | 88795 | 77 | cholesteryl | CE(20:1(11Z)) |
| 9 | 33667517 | fat cholesterol | 17002 | 76 | a cholesterol ester | cholesteryl ester |
| 10 | 33667517 | fat cholesterol | 17168 | 75 | gamma-cholesterol | 5alpha-cholest-7-en-3beta-ol |
| 11 | 33667517 | fat cholesterol | 64294 | 75 | 7-ketocholesterol | 7-ketocholesterol |
| 12 | 33667517 | fat cholesterol | 63906 | 74 | cholesterone | cholest-5-en-3-one |
| 13 | 33667517 | fat cholesterol | 77845 | 73 | 7-aminocholesterol | 7-aminocholesterol |
| 14 | 33667517 | fat cholesterol | 143264 | 73 | 22-ketocholesterol | 22-ketocholesterol |
| 15 | 33667517 | fat cholesterol | 17038 | 72 | fecosterol | fecosterol |
| 16 | 33667517 | fat cholesterol | 27865 | 72 | fucosterol | fucosterol |
| 17 | 33667517 | fat cholesterol | 63906 | 71 | oxidize cholesterol | cholest-5-en-3-one |
| 18 | 33667517 | fat cholesterol | 64294 | 71 | 7-oxocholesterol | 7-ketocholesterol |
| 19 | 33667517 | fat cholesterol | 79879 | 71 | b-norcholesterol | B-norcholesterol |
| 20 | 33667517 | fat cholesterol | 8427 | 70 | lesterol | probucol |
| 21 | 33667517 | fat cholesterol | 15889 | 70 | a sterol | sterol |
| 22 | 33667517 | fat cholesterol | 73085 | 70 | colterol | colterol |
| 23 | 33667517 | fat cholesterol | 6453 | 69 | lichesterol | Lichesterol |
| 24 | 33667517 | fat cholesterol | 16113 | 69 | cholesterin | cholesterol |
| 25 | 33667517 | fat cholesterol | 17002 | 69 | cholesterol ester | cholesteryl ester |
| 26 | 33667517 | fat cholesterol | 35821 | 69 | anticholesteremic | anticholesteremic drug |
| 27 | 33667517 | fat cholesterol | 46898 | 69 | oleoylcholesterol | cholesteryl oleate |
| 28 | 33667517 | fat cholesterol | 51277 | 69 | thiol ester | thioester |
| 29 | 33667517 | fat cholesterol | 61656 | 69 | glycosyl cholesterol | cholesteryl glycoside |
| 30 | 33667517 | fat cholesterol | 65640 | 69 | clathsterol | clathsterol |
| 31 | 33667517 | fat cholesterol | 66521 | 69 | stachsterol | stachsterol |
| 32 | 33667517 | fat cholesterol | 67554 | 69 | daucosterol | daucosterol |
| 33 | 33667517 | fat cholesterol | 86570 | 69 | cholestanol | (5alpha)-cholestan-3beta-ol |
| 34 | 33667517 | fat cholesterol | 135372 | 69 | cholostebol | clostebol |
| 35 | 33667517 | fat cholesterol | 143264 | 69 | 22-oxocholesterol | 22-ketocholesterol |
| 36 | 33667517 | fat cholesterol | 50404 | 68 | lipoprotein cholesterol | lipoprotein cholesterol |
| 37 | 33667517 | fat cholesterol | 132823 | 68 | 24beta-ethylcholesterol | clionasterol |
| 38 | 33667517 | fat cholesterol | 3641 | 67 | chlorestrolo | chlorotrianisene |
| 39 | 33667517 | fat cholesterol | 16175 | 67 | cholestenone | cholest-4-en-3-one |
| 40 | 33667517 | fat cholesterol | 27693 | 67 | 24alpha-ethylcholesterol | sitosterol |
| 41 | 33667517 | fat cholesterol | 34782 | 67 | hatomasterol | Hatomasterol |
| 42 | 33667517 | fat cholesterol | 35821 | 67 | antihypercholesterolemic | anticholesteremic drug |
| 43 | 33667517 | fat cholesterol | 37733 | 67 | anticholinesterase | EC 3.1.1.8 (cholinesterase) inhibitor |
| 44 | 33667517 | fat cholesterol | 46898 | 67 | cholesteryl oleate | cholesteryl oleate |
| 45 | 33667517 | fat cholesterol | 51277 | 67 | thioester | thioester |
| 46 | 33667517 | fat cholesterol | 71550 | 67 | lathosterone | lathosterone |
| 47 | 33667517 | fat cholesterol | 86570 | 67 | dihydrocholesterol | (5alpha)-cholestan-3beta-ol |
| 48 | 33667517 | fat cholesterol | 89281 | 67 | 7a-hydroxycholesterol | 7a-Hydroxycholesterol |
| 49 | 33667517 | fat cholesterol | 142773 | 67 | oestetrol | estetrol |
| 50 | 33667517 | fat cholesterol | 166835 | 67 | tachysterol3 | tachysterol 3 |
| 51 | 33667517 | fat cholesterol | 3663 | 65 | 16:0 cholesterol ester | cholesteryl palmitate |
| 52 | 33667517 | fat cholesterol | 19812 | 65 | 24-methylencholesterol | 24-methylenecholesterol |
| 53 | 33667517 | fat cholesterol | 23203 | 65 | cholestenoyl-coa | cholestenoyl-CoA |
| 54 | 33667517 | fat cholesterol | 35748 | 65 | fatty acid ester | fatty acid ester |
| 55 | 33667517 | fat cholesterol | 35821 | 65 | antihypercholesterolemics | anticholesteremic drug |
| 56 | 33667517 | fat cholesterol | 41321 | 65 | cholesterol sulfate | cholesterol sulfate |
| 57 | 33667517 | fat cholesterol | 47774 | 65 | beta-lipoprotein cholesterol | low-density lipoprotein cholesterol |
| 58 | 33667517 | fat cholesterol | 78206 | 65 | fatty acid ethyl ester | fatty acid ethyl ester |
| 59 | 33667517 | fat cholesterol | 78242 | 65 | cholesterol acetate | cholesteryl acetate |
| 60 | 33667517 | fat cholesterol | 88740 | 65 | 24:0 cholesterol ester | CE(24:0) |
| 61 | 33667517 | fat cholesterol | 88763 | 65 | 20:0 cholesterol ester | CE(20:0) |
| 62 | 33667517 | fat cholesterol | 88774 | 65 | 22:2 cholesterol ester | CE(22:2(13Z,16Z)) |
| 63 | 33667517 | fat cholesterol | 88775 | 65 | 20:2 cholesterol ester | CE(20:2(6Z,9Z)) |
| 64 | 33667517 | fat cholesterol | 88795 | 65 | 20:1 cholesterol ester | CE(20:1(11Z)) |
| 65 | 33667517 | fat cholesterol | 89029 | 65 | cholesterol caprate | CE(10:0) |
| 66 | 33667517 | fat cholesterol | 89281 | 65 | 7-a-hydroxycholesterol | 7a-Hydroxycholesterol |
| 67 | 33667517 | fat cholesterol | 133745 | 65 | 19:0 cholesterol ester | cholesteryl nonadecanoate |
| 68 | 33667517 | fat cholesterol | 3133 | 64 | bitolterol | bitolterol |
| 69 | 33667517 | fat cholesterol | 3814 | 64 | colestipol | colestipol |
| 70 | 33667517 | fat cholesterol | 3908 | 64 | coumestrol | coumestrol |
| 71 | 33667517 | fat cholesterol | 4532 | 64 | fosfestrol | diethylstilbestrol diphosphate |
| 72 | 33667517 | fat cholesterol | 7940 | 64 | patchoulol | patchouli alcohol |
| 73 | 33667517 | fat cholesterol | 9011 | 64 | salmeterol | salmeterol |
| 74 | 33667517 | fat cholesterol | 16521 | 64 | lanosterol | lanosterol |
| 75 | 33667517 | fat cholesterol | 19812 | 64 | chalinasterol | 24-methylenecholesterol |
| 76 | 33667517 | fat cholesterol | 23213 | 64 | choline ester | choline ester |
| 77 | 33667517 | fat cholesterol | 26819 | 64 | sulfate ester | sulfuric ester |
| 78 | 33667517 | fat cholesterol | 27693 | 64 | sitosterol | sitosterol |
| 79 | 33667517 | fat cholesterol | 28604 | 64 | isofucosterol | isofucosterol |
| 80 | 33667517 | fat cholesterol | 28940 | 64 | activate 7-dehydrocholesterol | calciol |
| 81 | 33667517 | fat cholesterol | 31578 | 64 | ethylestrenol | ethylestrenol |
| 82 | 33667517 | fat cholesterol | 31669 | 64 | hexoestrol | hexestrol |
| 83 | 33667517 | fat cholesterol | 34857 | 64 | moxesterol | Moxestrol |
| 84 | 33667517 | fat cholesterol | 35516 | 64 | cholestane | cholestane |
| 85 | 33667517 | fat cholesterol | 47775 | 64 | alpha-lipoprotein cholesterol | high-density lipoprotein cholesterol |
| 86 | 33667517 | fat cholesterol | 51277 | 64 | thioesters | thioester |
| 87 | 33667517 | fat cholesterol | 51277 | 64 | thio ester | thioester |
| 88 | 33667517 | fat cholesterol | 52343 | 64 | formate ester | formate ester |
| 89 | 33667517 | fat cholesterol | 71989 | 64 | orthoester | ortho ester |
| 90 | 33667517 | fat cholesterol | 73085 | 64 | colterolum | colterol |
| 91 | 33667517 | fat cholesterol | 131736 | 64 | cholane ester | cholane ester |
| 92 | 33667517 | fat cholesterol | 135146 | 64 | folescutol | folescutol |
| 93 | 33667517 | fat cholesterol | 135263 | 64 | methestrol | methestrol |
| 94 | 33667517 | fat cholesterol | 135264 | 64 | octoestrol | benzestrol |
| 95 | 33667517 | fat cholesterol | 135733 | 64 | thiomesterone | tiomesterone |
| 96 | 33667517 | fat cholesterol | 166835 | 64 | tachysterol 3 | tachysterol 3 |
| 97 | 33667517 | fat cholesterol | 4986 | 63 | fatty acid methyl ester | fatty acid methyl ester |
| 98 | 33667517 | fat cholesterol | 17759 | 63 | 7-dehydrocholesterol | cholesta-5,7-dien-3beta-ol |
| 99 | 33667517 | fat cholesterol | 19812 | 63 | 24-methylenecholesterol | 24-methylenecholesterol |
| 100 | 33667517 | fat cholesterol | 41321 | 63 | cholesterol sulphate | cholesterol sulfate |
| 101 | 33667517 | fat cholesterol | 42989 | 63 | 7-hydroxycholesterol | 7beta-hydroxycholesterol |
| 102 | 33667517 | fat cholesterol | 49305 | 63 | 5,6alpha epoxy-cholesterol | 5,6alpha-epoxy-5alpha-cholestan-3beta-ol |
| 103 | 33667517 | fat cholesterol | 82750 | 63 | cholesterol stearate | cholesteryl stearate |
| 104 | 33667517 | fat cholesterol | 84304 | 63 | 1-myristoyl-cholesterol | cholesteryl myristate |
| 105 | 33667517 | fat cholesterol | 84323 | 63 | 1-palmitoleoyl-cholesterol | cholesteryl palmitoleate |
| 106 | 33667517 | fat cholesterol | 89982 | 63 | 8-dehydrocholesterol | 8-Dehydrocholesterol |
| 107 | 33667517 | fat cholesterol | 133745 | 63 | nonadecanoylcholesterol | cholesteryl nonadecanoate |
| 108 | 33667517 | fat cholesterol | 177021 | 63 | fatty acid phytyl ester | fatty acid phytyl ester |
| 109 | 33667517 | fat cholesterol | 6836 | 62 | methostenol | Methostenol |
| 110 | 33667517 | fat cholesterol | 17002 | 62 | cholesteryl ester | cholesteryl ester |
| 111 | 33667517 | fat cholesterol | 23203 | 62 | cholestenoyl-coas | cholestenoyl-CoA |
| 112 | 33667517 | fat cholesterol | 23990 | 62 | ethyl ester | ethyl ester |
| 113 | 33667517 | fat cholesterol | 26925 | 62 | naphthalenetetrol | naphthalenetetrol |
| 114 | 33667517 | fat cholesterol | 27173 | 62 | typhasterol | typhasterol |
| 115 | 33667517 | fat cholesterol | 28623 | 62 | campesterol | campesterol |
| 116 | 33667517 | fat cholesterol | 28824 | 62 | phytosterol | stigmasterol |
| 117 | 33667517 | fat cholesterol | 31542 | 62 | epicholestanol | epidihydrocholesterin |
| 118 | 33667517 | fat cholesterol | 31669 | 62 | cycloestrol | hexestrol |
| 119 | 33667517 | fat cholesterol | 31669 | 62 | hormoestrol | hexestrol |
| 120 | 33667517 | fat cholesterol | 32131 | 62 | cholesolvin | Simfibrate |
| 121 | 33667517 | fat cholesterol | 32402 | 62 | catecholate | catecholate(2-) |
| 122 | 33667517 | fat cholesterol | 34749 | 62 | aethisteron | ethisterone |
| 123 | 33667517 | fat cholesterol | 35915 | 62 | a sterol ester | sterol ester |
| 124 | 33667517 | fat cholesterol | 42989 | 62 | 7beta-hydroxycholesterol | 7beta-hydroxycholesterol |
| 125 | 33667517 | fat cholesterol | 48977 | 62 | fast scarlet r | 2-methoxy-5-nitroaniline |
| 126 | 33667517 | fat cholesterol | 51278 | 62 | thionoester | thionoester |
| 127 | 33667517 | fat cholesterol | 52473 | 62 | triflate ester | triflate ester |
| 128 | 33667517 | fat cholesterol | 59785 | 62 | dithioester | dithioester |
| 129 | 33667517 | fat cholesterol | 61829 | 62 | colestolone | 3beta-hydroxy-5alpha-cholest-8(14)-en-15-one |
| 130 | 33667517 | fat cholesterol | 61829 | 62 | colestolona | 3beta-hydroxy-5alpha-cholest-8(14)-en-15-one |
| 131 | 33667517 | fat cholesterol | 64292 | 62 | a coniferyl ester | coniferyl ester |
| 132 | 33667517 | fat cholesterol | 66571 | 62 | leucisterol | leucisterol |
| 133 | 33667517 | fat cholesterol | 71989 | 62 | ortho ester | ortho ester |
| 134 | 33667517 | fat cholesterol | 71989 | 62 | orthoesters | ortho ester |
| 135 | 33667517 | fat cholesterol | 71989 | 62 | ortho-ester | ortho ester |
| 136 | 33667517 | fat cholesterol | 78206 | 62 | a fatty acid ethyl ester | fatty acid ethyl ester |
| 137 | 33667517 | fat cholesterol | 85778 | 62 | 4beta-hydroxycholesterol | 4beta-hydroxycholesterol |
| 138 | 33667517 | fat cholesterol | 89519 | 62 | coprosterol | coprostanol |
| 139 | 33667517 | fat cholesterol | 90106 | 62 | fast violet | gallocyanin |
| 140 | 33667517 | fat cholesterol | 131643 | 62 | ketocholestane | ketocholestane |
| 141 | 33667517 | fat cholesterol | 135146 | 62 | pholescutol | folescutol |
| 142 | 33667517 | fat cholesterol | 137062 | 62 | 7-ketocholestanol | 7-oxo-5alpha-cholestan-3beta-ol |
| 143 | 33667517 | fat cholesterol | 138486 | 62 | cholest-5-en-3-ol | cholest-5-en-3-ol |
| 144 | 33667517 | fat cholesterol | 155905 | 62 | crinosterol | crinosterol |
| 145 | 33667517 | fat cholesterol | 166888 | 62 | avenasterol | avenasterol |
| 146 | 33667517 | fat cholesterol | 174690 | 62 | clenbuterol | clenbuterol |
| 147 | 33667517 | fat cholesterol | 1296 | 61 | 20-hydroxycholesterol | 20-hydroxycholesterol |
| 148 | 33667517 | fat cholesterol | 2565 | 61 | alectrol | Alectrol |
| 149 | 33667517 | fat cholesterol | 3663 | 61 | cholesterol palmitate | cholesteryl palmitate |
| 150 | 33667517 | fat cholesterol | 17495 | 61 | cholesterol glucoside | cholesteryl beta-D-glucoside |
| 151 | 33667517 | fat cholesterol | 17703 | 61 | 26-hydroxycholesterol | 26-hydroxycholesterol |
| 152 | 33667517 | fat cholesterol | 17703 | 61 | 27-hydroxycholesterol | 26-hydroxycholesterol |
| 153 | 33667517 | fat cholesterol | 17737 | 61 | 24-dehydrocholesterol | desmosterol |
| 154 | 33667517 | fat cholesterol | 27974 | 61 | oestriol | estriol |
| 155 | 33667517 | fat cholesterol | 33308 | 61 | a carboxylic ester | carboxylic ester |
| 156 | 33667517 | fat cholesterol | 34310 | 61 | 24-hydroxycholesterol | (24S)-24-hydroxycholesterol |
| 157 | 33667517 | fat cholesterol | 35748 | 61 | a fatty acid ester | fatty acid ester |
| 158 | 33667517 | fat cholesterol | 35821 | 61 | cholesterol inhibitor | anticholesteremic drug |
| 159 | 33667517 | fat cholesterol | 41321 | 61 | cholesterol 3-sulfate | cholesterol sulfate |
| 160 | 33667517 | fat cholesterol | 41509 | 61 | cholesteryl linoleate | cholesteryl linoleate |
| 161 | 33667517 | fat cholesterol | 42977 | 61 | 25-hydroxycholesterol | 25-hydroxycholesterol |
| 162 | 33667517 | fat cholesterol | 52321 | 61 | a fecosterol ester | fecosterol ester |
| 163 | 33667517 | fat cholesterol | 61656 | 61 | cholesterol glycoside | cholesteryl glycoside |
| 164 | 33667517 | fat cholesterol | 78242 | 61 | cholesterol 3-acetate | cholesteryl acetate |
| 165 | 33667517 | fat cholesterol | 80797 | 61 | hysterol | Bornyl isovalerate |
| 166 | 33667517 | fat cholesterol | 86570 | 61 | 5alpha-cholestanol | (5alpha)-cholestan-3beta-ol |
| 167 | 33667517 | fat cholesterol | 88753 | 61 | 1-erucoyl-cholesterol | CE(22:1(13Z)) |
| 168 | 33667517 | fat cholesterol | 89029 | 61 | cholesterol decanoate | CE(10:0) |
| 169 | 33667517 | fat cholesterol | 135998 | 61 | iodocholesterol i 131 | iodocholesterol (131I) |
| 170 | 33667517 | fat cholesterol | 142773 | 61 | estetrol | estetrol |
| 171 | 33667517 | fat cholesterol | 4986 | 60 | a fatty acid methyl ester | fatty acid methyl ester |
| 172 | 33667517 | fat cholesterol | 6808 | 60 | methallenestrol | Methallenestril |
| 173 | 33667517 | fat cholesterol | 17500 | 60 | 7alpha-hydroxycholesterol | 7alpha-hydroxycholesterol |
| 174 | 33667517 | fat cholesterol | 27693 | 60 | beta-sitosterol | sitosterol |
| 175 | 33667517 | fat cholesterol | 28164 | 60 | 5,6beta-epoxy-cholesterol | cholesterol beta-epoxide |
| 176 | 33667517 | fat cholesterol | 28940 | 60 | cholecalciferol | calciol |
| 177 | 33667517 | fat cholesterol | 31542 | 60 | epi-cholestanol | epidihydrocholesterin |
| 178 | 33667517 | fat cholesterol | 31578 | 60 | ethylestrenolum | ethylestrenol |
| 179 | 33667517 | fat cholesterol | 31790 | 60 | ethynylestrenol | Lynestrenol |
| 180 | 33667517 | fat cholesterol | 35517 | 60 | beta-cholestane | 5beta-cholestane |
| 181 | 33667517 | fat cholesterol | 47622 | 60 | an acetyl ester | acetate ester |
| 182 | 33667517 | fat cholesterol | 47773 | 60 | pre-beta-lipoprotein cholesterol | very-low-density lipoprotein cholesterol |
| 183 | 33667517 | fat cholesterol | 48199 | 60 | sulfamate ester | sulfamate ester |
| 184 | 33667517 | fat cholesterol | 50856 | 60 | 2-furoate ester | 2-furoate ester |
| 185 | 33667517 | fat cholesterol | 52474 | 60 | sulfonate ester | sulfonate ester |
| 186 | 33667517 | fat cholesterol | 67237 | 60 | 22beta-hydroxycholesterol | (22R)-22-hydroxycholesterol |
| 187 | 33667517 | fat cholesterol | 84883 | 60 | epa ethyl ester | ethyl (5Z,8Z,11Z,14Z,17Z)-icosapentaenoate |
| 188 | 33667517 | fat cholesterol | 84945 | 60 | dha ethyl ester | ethyl (4Z,7Z,10Z,13Z,16Z,19Z)-docosahexaenoate |
| 189 | 33667517 | fat cholesterol | 88763 | 60 | 1-arachidonyl-cholesterol | CE(20:0) |
| 190 | 33667517 | fat cholesterol | 131643 | 60 | ketocholestanes | ketocholestane |
| 191 | 33667517 | fat cholesterol | 142575 | 60 | 25-acyl-27-norcholesterol | 25-acyl-27-norcholesterol |
| 192 | 33667517 | fat cholesterol | 177021 | 60 | a fatty acid phytyl ester | fatty acid phytyl ester |
| 193 | 33667517 | fat cholesterol | 1296 | 59 | 20alpha-hydroxycholesterol | 20-hydroxycholesterol |
| 194 | 33667517 | fat cholesterol | 3133 | 59 | bitolterolum | bitolterol |
| 195 | 33667517 | fat cholesterol | 3814 | 59 | colestipolum | colestipol |
| 196 | 33667517 | fat cholesterol | 4532 | 59 | phosphestrol | diethylstilbestrol diphosphate |
| 197 | 33667517 | fat cholesterol | 4532 | 59 | stilphostrol | diethylstilbestrol diphosphate |
| 198 | 33667517 | fat cholesterol | 6347 | 59 | lachnophyllum ester | Lachnophyllum ester |
| 199 | 33667517 | fat cholesterol | 6570 | 59 | fagarasterol | lupeol |
| 200 | 33667517 | fat cholesterol | 7940 | 59 | patchoulanol | patchouli alcohol |
| 201 | 33667517 | fat cholesterol | 9011 | 59 | salmeterolum | salmeterol |
| 202 | 33667517 | fat cholesterol | 17500 | 59 | 7alpha-hydroxy-cholesterol | 7alpha-hydroxycholesterol |
| 203 | 33667517 | fat cholesterol | 19812 | 59 | ostreasterol | 24-methylenecholesterol |
| 204 | 33667517 | fat cholesterol | 23057 | 59 | cathasterone | cathasterone |
| 205 | 33667517 | fat cholesterol | 23908 | 59 | eleostearoyl | eleostearoyl group |
| 206 | 33667517 | fat cholesterol | 25248 | 59 | methyl ester | methyl ester |
| 207 | 33667517 | fat cholesterol | 26125 | 59 | phytosterols | phytosterols |
| 208 | 33667517 | fat cholesterol | 27750 | 59 | acetic ester | ethyl acetate |
| 209 | 33667517 | fat cholesterol | 27750 | 59 | acetyl ester | ethyl acetate |
| 210 | 33667517 | fat cholesterol | 28810 | 59 | 5-cholestene | cholest-5-ene |
| 211 | 33667517 | fat cholesterol | 28934 | 59 | activate ergosterol | vitamin D2 |
| 212 | 33667517 | fat cholesterol | 30408 | 59 | fe-s cluster | iron-sulfur cluster |
| 213 | 33667517 | fat cholesterol | 31578 | 59 | etilestrenol | ethylestrenol |
| 214 | 33667517 | fat cholesterol | 31669 | 59 | hexanoestrol | hexestrol |
| 215 | 33667517 | fat cholesterol | 34310 | 59 | 24s-hydroxycholesterol | (24S)-24-hydroxycholesterol |
| 216 | 33667517 | fat cholesterol | 35821 | 59 | anticholesteremic drug | anticholesteremic drug |
| 217 | 33667517 | fat cholesterol | 35821 | 59 | antihypercholesterolemic drug | anticholesteremic drug |
| 218 | 33667517 | fat cholesterol | 35915 | 59 | sterol ester | sterol ester |
| 219 | 33667517 | fat cholesterol | 36188 | 59 | malate ester | malate ester |
| 220 | 33667517 | fat cholesterol | 38915 | 59 | borate ester | borate esters |
| 221 | 33667517 | fat cholesterol | 41321 | 59 | cholesteryl sulfate | cholesterol sulfate |
| 222 | 33667517 | fat cholesterol | 41321 | 59 | cholesterol 3-sulphate | cholesterol sulfate |
| 223 | 33667517 | fat cholesterol | 41509 | 59 | 18:2 cholesteryl ester | cholesteryl linoleate |
| 224 | 33667517 | fat cholesterol | 46898 | 59 | 18:1 cholesteryl ester | cholesteryl oleate |
| 225 | 33667517 | fat cholesterol | 50401 | 59 | cholestanoid | cholestanoid |
| 226 | 33667517 | fat cholesterol | 50516 | 59 | 24r-hydroxycholesterol | (24R)-24-hydroxycholesterol |
| 227 | 33667517 | fat cholesterol | 51278 | 59 | thionoesters | thionoester |
| 228 | 33667517 | fat cholesterol | 51702 | 59 | enoate ester | enoate ester |
| 229 | 33667517 | fat cholesterol | 51743 | 59 | ynoate ester | ynoate ester |
| 230 | 33667517 | fat cholesterol | 52343 | 59 | formyl ester | formate ester |
| 231 | 33667517 | fat cholesterol | 59785 | 59 | dithio ester | dithioester |
| 232 | 33667517 | fat cholesterol | 59785 | 59 | dithioesters | dithioester |
| 233 | 33667517 | fat cholesterol | 61829 | 59 | colestolonum | 3beta-hydroxy-5alpha-cholest-8(14)-en-15-one |
| 234 | 33667517 | fat cholesterol | 65998 | 59 | halosterol a | halosterol A |
| 235 | 33667517 | fat cholesterol | 65999 | 59 | halosterol b | halosterol B |
| 236 | 33667517 | fat cholesterol | 67237 | 59 | 22r-hydroxycholesterol | (22R)-22-hydroxycholesterol |
| 237 | 33667517 | fat cholesterol | 68083 | 59 | cerevisterol | cerevisterol |
| 238 | 33667517 | fat cholesterol | 71989 | 59 | ortho-esters | ortho ester |
| 239 | 33667517 | fat cholesterol | 74102 | 59 | 22:6 cholesteryl ester | cholesteryl (4Z,7Z,10Z,13Z,16Z,19Z)-docosahexaenoate |
| 240 | 33667517 | fat cholesterol | 74103 | 59 | cholesterol ester 22:6 | CE(22:6) |
| 241 | 33667517 | fat cholesterol | 74537 | 59 | thioglycerol | monothioglycerol |
| 242 | 33667517 | fat cholesterol | 78242 | 59 | cholesteryl acetate | cholesteryl acetate |
| 243 | 33667517 | fat cholesterol | 82750 | 59 | 18:0 cholesteryl ester | cholesteryl stearate |
| 244 | 33667517 | fat cholesterol | 82751 | 59 | 20:4 cholesteryl ester | cholesteryl arachidonate |
| 245 | 33667517 | fat cholesterol | 84318 | 59 | 15:0 cholesteryl ester | cholesteryl pentadecanoate |
| 246 | 33667517 | fat cholesterol | 84326 | 59 | 17:0 cholesteryl ester | cholesteryl heptadecanoate |
| 247 | 33667517 | fat cholesterol | 84341 | 59 | cholesteryl linolenate | cholesteryl linolenate |
| 248 | 33667517 | fat cholesterol | 84341 | 59 | 18:3 cholesteryl ester | cholesteryl linolenate |
| 249 | 33667517 | fat cholesterol | 84346 | 59 | 20:3 cholesteryl ester | cholesteryl all-cis-icosa-8,11,14-trienoate |
| 250 | 33667517 | fat cholesterol | 84352 | 59 | 22:0 cholesteryl ester | cholesteryl behenate |
| 251 | 33667517 | fat cholesterol | 88753 | 59 | cholesterol 1-erucoate | CE(22:1(13Z)) |
| 252 | 33667517 | fat cholesterol | 88755 | 59 | cholesterol 1-eicsoate | CE(20:4(8Z,11Z,14Z,17Z)) |
| 253 | 33667517 | fat cholesterol | 88758 | 59 | 1-myristoleoyl-cholesterol | CE(14:1(9Z)) |
| 254 | 33667517 | fat cholesterol | 88763 | 59 | cholesterol arachidate | CE(20:0) |
| 255 | 33667517 | fat cholesterol | 89029 | 59 | cholesteryl caprate | CE(10:0) |
| 256 | 33667517 | fat cholesterol | 89029 | 59 | cholesterol n-decylate | CE(10:0) |
| 257 | 33667517 | fat cholesterol | 89281 | 59 | 7 alpha-hydroxycholesterol | 7a-Hydroxycholesterol |
| 258 | 33667517 | fat cholesterol | 89281 | 59 | 7-alpha-hydroxycholesterol | 7a-Hydroxycholesterol |
| 259 | 33667517 | fat cholesterol | 132823 | 59 | clionasterol | clionasterol |
| 260 | 33667517 | fat cholesterol | 133745 | 59 | 19:0 cholesteryl ester | cholesteryl nonadecanoate |
| 261 | 33667517 | fat cholesterol | 135733 | 59 | tiomesterone | tiomesterone |
| 262 | 33667517 | fat cholesterol | 135733 | 59 | thiomestrone | tiomesterone |
| 263 | 33667517 | fat cholesterol | 137551 | 59 | cholesterol ester 22:5 | CE(22:5) |
| 264 | 33667517 | fat cholesterol | 137552 | 59 | cholesterol ester 16:1 | CE(16:1) |
| 265 | 33667517 | fat cholesterol | 137554 | 59 | cholesterol ester 17:1 | CE(17:1) |
| 266 | 33667517 | fat cholesterol | 137555 | 59 | cholesterol ester 17:2 | CE(17:2) |
| 267 | 33667517 | fat cholesterol | 137556 | 59 | cholesterol ester 19:2 | CE(19:2) |
| 268 | 33667517 | fat cholesterol | 137557 | 59 | cholesterol ester 19:3 | CE(19:3) |
| 269 | 33667517 | fat cholesterol | 142575 | 59 | 25-substituted-cholesterol | 25-acyl-27-norcholesterol |
| 270 | 33667517 | fat cholesterol | 142698 | 59 | 22:4 cholesteryl ester | CE(22:4(7Z,10Z,13Z,16Z)) |
| 271 | 33667517 | fat cholesterol | 2549 | 58 | albuterol | albuterol |
| 272 | 33667517 | fat cholesterol | 4903 | 58 | ethinylestradiol | 17alpha-ethynylestradiol |
| 273 | 33667517 | fat cholesterol | 10036 | 58 | wax ester | wax ester |
| 274 | 33667517 | fat cholesterol | 10055 | 58 | xamoterol | Xamoterol |
| 275 | 33667517 | fat cholesterol | 16385 | 58 | thioether | organic sulfide |
| 276 | 33667517 | fat cholesterol | 23197 | 58 | cholestanoyl-coa | cholestanoyl-CoA |
| 277 | 33667517 | fat cholesterol | 23929 | 58 | episterol | episterol |
| 278 | 33667517 | fat cholesterol | 26178 | 58 | polyester | polyester macromolecule |
| 279 | 33667517 | fat cholesterol | 27013 | 58 | tocoferol | tocopherol |
| 280 | 33667517 | fat cholesterol | 27118 | 58 | naphthalenetriol | naphthalenetriol |
| 281 | 33667517 | fat cholesterol | 27750 | 58 | acetic acid ethyl ester | ethyl acetate |
| 282 | 33667517 | fat cholesterol | 27865 | 58 | trans-24-ethylidenecholesterol | fucosterol |
| 283 | 33667517 | fat cholesterol | 28540 | 58 | cholestane-3,7,26-triol | 5beta-cholestane-3alpha,7alpha,26-triol |
| 284 | 33667517 | fat cholesterol | 28604 | 58 | 28-isofucosterol | isofucosterol |
| 285 | 33667517 | fat cholesterol | 28934 | 58 | viosterol | vitamin D2 |
| 286 | 33667517 | fat cholesterol | 31669 | 58 | hexestrol | hexestrol |
| 287 | 33667517 | fat cholesterol | 31669 | 58 | erythrohexestrol | hexestrol |
| 288 | 33667517 | fat cholesterol | 33566 | 58 | catechols | catechols |
| 289 | 33667517 | fat cholesterol | 34310 | 58 | 24s-hydroxy-cholesterol | (24S)-24-hydroxycholesterol |
| 290 | 33667517 | fat cholesterol | 34857 | 58 | moxestrol | Moxestrol |
| 291 | 33667517 | fat cholesterol | 35341 | 58 | a steroid | steroid |
| 292 | 33667517 | fat cholesterol | 35515 | 58 | alpha-cholestane | 5alpha-cholestane |
| 293 | 33667517 | fat cholesterol | 35517 | 58 | 5beta-cholestane | 5beta-cholestane |
| 294 | 33667517 | fat cholesterol | 35821 | 58 | anticholesteremic agent | anticholesteremic drug |
| 295 | 33667517 | fat cholesterol | 35821 | 58 | antihypercholesterolemic agent | anticholesteremic drug |
| 296 | 33667517 | fat cholesterol | 41633 | 58 | 24,25-epoxy-cholesterol | 24(S),25-epoxycholesterol |
| 297 | 33667517 | fat cholesterol | 46831 | 58 | naphthoate ester | naphthoate ester |
| 298 | 33667517 | fat cholesterol | 49305 | 58 | cholesterol alpha-oxide | 5,6alpha-epoxy-5alpha-cholestan-3beta-ol |
| 299 | 33667517 | fat cholesterol | 51847 | 58 | ketoester | ketoester |
| 300 | 33667517 | fat cholesterol | 52321 | 58 | fecosterol ester | fecosterol ester |
| 301 | 33667517 | fat cholesterol | 52394 | 58 | lanosterol ester | lanosteryl ester |
| 302 | 33667517 | fat cholesterol | 53030 | 58 | oxysterol | oxysterol |
| 303 | 33667517 | fat cholesterol | 53615 | 58 | manchester brown | Bismark brown Y |
| 304 | 33667517 | fat cholesterol | 63959 | 58 | celastrol | celastrol |
| 305 | 33667517 | fat cholesterol | 67554 | 58 | beta-daucosterol | daucosterol |
| 306 | 33667517 | fat cholesterol | 68505 | 58 | catechol sulfate | pyrocatechol sulfate |
| 307 | 33667517 | fat cholesterol | 73250 | 58 | ga1 methyl ester | gibberellin A1 methyl ester |
| 308 | 33667517 | fat cholesterol | 73252 | 58 | ga4 methyl ester | gibberellin A4 methyl ester |
| 309 | 33667517 | fat cholesterol | 73253 | 58 | ga3 methyl ester | gibberellin A3 methyl ester |
| 310 | 33667517 | fat cholesterol | 73256 | 58 | ga9 methyl ester | gibberellin A9 methyl ester |
| 311 | 33667517 | fat cholesterol | 78319 | 58 | lactic acid ethyl ester | rac-ethyl lactate |
| 312 | 33667517 | fat cholesterol | 79728 | 58 | 3-methoxyestriol | 3-Methoxyestriol |
| 313 | 33667517 | fat cholesterol | 80094 | 58 | 4alpha-methylfecosterol | 4alpha-Methylfecosterol |
| 314 | 33667517 | fat cholesterol | 81307 | 58 | 7-oxatyphasterol | 7-Oxatyphasterol |
| 315 | 33667517 | fat cholesterol | 85715 | 58 | l cluster | L cluster |
| 316 | 33667517 | fat cholesterol | 87511 | 58 | acetic acid nonyl ester | nonyl acetate |
| 317 | 33667517 | fat cholesterol | 88754 | 58 | 1-docosapentaenoyl-cholesterol | CE(22:5(4Z,7Z,10Z,13Z,16Z)) |
| 318 | 33667517 | fat cholesterol | 88754 | 58 | 1-osbondoyl-cholesterol | CE(22:5(4Z,7Z,10Z,13Z,16Z)) |
| 319 | 33667517 | fat cholesterol | 88755 | 58 | 1-eicsoatetraenoyl-cholesterol | CE(20:4(8Z,11Z,14Z,17Z)) |
| 320 | 33667517 | fat cholesterol | 88768 | 58 | ( cis-vaccenoyl ) -cholesterol | (11Z-octadecenoyl)-cholesterol |
| 321 | 33667517 | fat cholesterol | 89029 | 58 | cholesterol 3-decanoate | CE(10:0) |
| 322 | 33667517 | fat cholesterol | 89234 | 58 | cholenate | 3b-Hydroxy-5-cholenoic acid |
| 323 | 33667517 | fat cholesterol | 131637 | 58 | lanostane sterol | lanostane sterol |
| 324 | 33667517 | fat cholesterol | 131696 | 58 | cholestane ester | cholestane ester |
| 325 | 33667517 | fat cholesterol | 132823 | 58 | gamma-sitosterol | clionasterol |
| 326 | 33667517 | fat cholesterol | 134476 | 58 | acetic acid oleyl ester | oleyl acetate |
| 327 | 33667517 | fat cholesterol | 134976 | 58 | arcaterol | xenbucin |
| 328 | 33667517 | fat cholesterol | 135135 | 58 | acetarsol | acetarsol |
| 329 | 33667517 | fat cholesterol | 135264 | 58 | octestrol | benzestrol |
| 330 | 33667517 | fat cholesterol | 135372 | 58 | clostebol | clostebol |
| 331 | 33667517 | fat cholesterol | 135751 | 58 | tobuterol | tobuterol |
| 332 | 33667517 | fat cholesterol | 137062 | 58 | 7-oxocholestanol | 7-oxo-5alpha-cholestan-3beta-ol |
| 333 | 33667517 | fat cholesterol | 149226 | 58 | fenoterol | fenoterol |
| 334 | 33667517 | fat cholesterol | 167166 | 58 | 3-ketocholestane | 3-oxocholestane |
| 335 | 33667517 | fat cholesterol | 3663 | 57 | palmitic acid cholesteryl ester | cholesteryl palmitate |
| 336 | 33667517 | fat cholesterol | 5861 | 57 | ichthyotherol | Ichthyotherol |
| 337 | 33667517 | fat cholesterol | 15889 | 57 | sterol | sterol |
| 338 | 33667517 | fat cholesterol | 23178 | 57 | cholest-7-ene | cholest-7-ene |
| 339 | 33667517 | fat cholesterol | 24026 | 57 | fatty alcohol | fatty alcohol |
| 340 | 33667517 | fat cholesterol | 27957 | 57 | acetol | hydroxyacetone |
| 341 | 33667517 | fat cholesterol | 28810 | 57 | cholest-5-ene | cholest-5-ene |
| 342 | 33667517 | fat cholesterol | 31189 | 57 | allylestrenol | Allylestrenol |
| 343 | 33667517 | fat cholesterol | 33573 | 57 | tetrol | tetrol |
| 344 | 33667517 | fat cholesterol | 34310 | 57 | cerebrosterol | (24S)-24-hydroxycholesterol |
| 345 | 33667517 | fat cholesterol | 34310 | 57 | ( 24s ) -hydroxycholesterol | (24S)-24-hydroxycholesterol |
| 346 | 33667517 | fat cholesterol | 34749 | 57 | aethisteronum | ethisterone |
| 347 | 33667517 | fat cholesterol | 34983 | 57 | stylisterol a | Stylisterol A |
| 348 | 33667517 | fat cholesterol | 34984 | 57 | stylisterol b | Stylisterol B |
| 349 | 33667517 | fat cholesterol | 34985 | 57 | stylisterol c | Stylisterol C |
| 350 | 33667517 | fat cholesterol | 35347 | 57 | 3alpha-sterol | 3alpha-sterol |
| 351 | 33667517 | fat cholesterol | 35486 | 57 | maleate ester | maleate ester |
| 352 | 33667517 | fat cholesterol | 36832 | 57 | cyanate ester | cyanate ester |
| 353 | 33667517 | fat cholesterol | 37532 | 57 | phorbol ester | phorbol ester |
| 354 | 33667517 | fat cholesterol | 37576 | 57 | gallate ester | gallate ester |
| 355 | 33667517 | fat cholesterol | 39088 | 57 | sulfite ester | sulfite ester |
| 356 | 33667517 | fat cholesterol | 41321 | 57 | cholesteryl sulphate | cholesterol sulfate |
| 357 | 33667517 | fat cholesterol | 46902 | 57 | cholesteryl elaidate | cholesteryl elaidate |
| 358 | 33667517 | fat cholesterol | 47622 | 57 | acetate ester | acetate ester |
| 359 | 33667517 | fat cholesterol | 50401 | 57 | cholestanoids | cholestanoid |
| 360 | 33667517 | fat cholesterol | 50516 | 57 | ( 24r ) -hydroxycholesterol | (24R)-24-hydroxycholesterol |
| 361 | 33667517 | fat cholesterol | 50751 | 57 | antioestrogen | anti-estrogen |
| 362 | 33667517 | fat cholesterol | 51080 | 57 | nitrate ester | nitrate ester |
| 363 | 33667517 | fat cholesterol | 61313 | 57 | a c21-steroid | C21-steroid |
| 364 | 33667517 | fat cholesterol | 62204 | 57 | coa thioether | S-alkyl-CoA |
| 365 | 33667517 | fat cholesterol | 66261 | 57 | trichodimerol | trichodimerol |
| 366 | 33667517 | fat cholesterol | 68082 | 57 | ganodesterone | ganodesterone |
| 367 | 33667517 | fat cholesterol | 70016 | 57 | cyathisterone | (22E,24R)-ergosta-7,22-diene-3,6-dione |
| 368 | 33667517 | fat cholesterol | 81215 | 57 | chrysantherol | Chrysantherol |
| 369 | 33667517 | fat cholesterol | 82750 | 57 | cholesteryl stearate | cholesteryl stearate |
| 370 | 33667517 | fat cholesterol | 83219 | 57 | lactate ester | lactate ester |
| 371 | 33667517 | fat cholesterol | 84352 | 57 | cholesteryl behenate | cholesteryl behenate |
| 372 | 33667517 | fat cholesterol | 87658 | 57 | caprate ester | decanoate ester |
| 373 | 33667517 | fat cholesterol | 87659 | 57 | laurate ester | dodecanoate ester |
| 374 | 33667517 | fat cholesterol | 88768 | 57 | 1-cis-vaccenoyl-cholesterol | (11Z-octadecenoyl)-cholesterol |
| 375 | 33667517 | fat cholesterol | 89029 | 57 | cholesteryl decylate | CE(10:0) |
| 376 | 33667517 | fat cholesterol | 131643 | 57 | oxocholestane | ketocholestane |
| 377 | 33667517 | fat cholesterol | 132468 | 57 | bacteriohopanetetrol | bacteriohopane-32,33,34,35-tetrol |
| 378 | 33667517 | fat cholesterol | 134308 | 57 | xanthogalenol | xanthogalenol(1-) |
| 379 | 33667517 | fat cholesterol | 135263 | 57 | promethestrol | methestrol |
| 380 | 33667517 | fat cholesterol | 139291 | 57 | a chlorophyll | chlorophyll(1-) |
| 381 | 33667517 | fat cholesterol | 142698 | 57 | cholesteryl adrenate | CE(22:4(7Z,10Z,13Z,16Z)) |
| 382 | 33667517 | fat cholesterol | 145763 | 57 | 6-ketoestriol | 6-ketoestriol |
| 383 | 33667517 | fat cholesterol | 174690 | 57 | clenbuterolum | clenbuterol |
| 384 | 33667517 | fat cholesterol | 177027 | 57 | laurate phytyl ester | laurate phytyl ester |
| 385 | 33667517 | fat cholesterol | 177028 | 57 | caprate phytyl ester | caprate phytyl ester |
| 386 | 33667517 | fat cholesterol | 1296 | 56 | 20 ( s ) -hydroxycholesterol | 20-hydroxycholesterol |
| 387 | 33667517 | fat cholesterol | 2906 | 56 | atheroline | Atheroline |
| 388 | 33667517 | fat cholesterol | 3663 | 56 | cholesteryl palmitate | cholesteryl palmitate |
| 389 | 33667517 | fat cholesterol | 4896 | 56 | pear ester | ethyl (2E,4Z)-deca-2,4-dienoate |
| 390 | 33667517 | fat cholesterol | 4903 | 56 | ethinyl estradiol | 17alpha-ethynylestradiol |
| 391 | 33667517 | fat cholesterol | 4903 | 56 | ethynyl estradiol | 17alpha-ethynylestradiol |
| 392 | 33667517 | fat cholesterol | 4903 | 56 | ethinyloestradiol | 17alpha-ethynylestradiol |
| 393 | 33667517 | fat cholesterol | 5147 | 56 | formoterol | formoterol |
| 394 | 33667517 | fat cholesterol | 6808 | 56 | vallestril | Methallenestril |
| 395 | 33667517 | fat cholesterol | 6949 | 56 | miroestrol | Miroestrol |
| 396 | 33667517 | fat cholesterol | 8463 | 56 | naphthylisoproterenol | Pronethalol |
| 397 | 33667517 | fat cholesterol | 9049 | 56 | schottenol | Schottenol |
| 398 | 33667517 | fat cholesterol | 16077 | 56 | cori ester | D-glucopyranose 1-phosphate |
| 399 | 33667517 | fat cholesterol | 16113 | 56 | cholest-5-en-3beta-ol | cholesterol |
| 400 | 33667517 | fat cholesterol | 16175 | 56 | 4-cholesten-3-one | cholest-4-en-3-one |
| 401 | 33667517 | fat cholesterol | 16359 | 56 | cholsaeure | cholic acid |
| 402 | 33667517 | fat cholesterol | 16385 | 56 | thioethers | organic sulfide |
| 403 | 33667517 | fat cholesterol | 16469 | 56 | 17beta oestradiol | 17beta-estradiol |
| 404 | 33667517 | fat cholesterol | 16514 | 56 | chryseriol | 4',5,7-trihydroxy-3'-methoxyflavone |
| 405 | 33667517 | fat cholesterol | 16933 | 56 | ergosterol | ergosterol |
| 406 | 33667517 | fat cholesterol | 17495 | 56 | cholesteryl glucoside | cholesteryl beta-D-glucoside |
| 407 | 33667517 | fat cholesterol | 17823 | 56 | decostriol | calcitriol |
| 408 | 33667517 | fat cholesterol | 18252 | 56 | zymosterol | zymosterol |
| 409 | 33667517 | fat cholesterol | 23197 | 56 | cholestanoyl-coas | cholestanoyl-CoA |
| 410 | 33667517 | fat cholesterol | 23965 | 56 | oestradiol | estradiol |
| 411 | 33667517 | fat cholesterol | 26863 | 56 | teasterone | teasterone |
| 412 | 33667517 | fat cholesterol | 27013 | 56 | tocopherol | tocopherol |
| 413 | 33667517 | fat cholesterol | 27865 | 56 | fucosterin | fucosterol |
| 414 | 33667517 | fat cholesterol | 28113 | 56 | lanostenol | 24,25-dihydrolanosterol |
| 415 | 33667517 | fat cholesterol | 28164 | 56 | cholesterol beta-epoxide | cholesterol beta-epoxide |
| 416 | 33667517 | fat cholesterol | 31428 | 56 | colestilan | Colestilan |
| 417 | 33667517 | fat cholesterol | 31588 | 56 | fabesetron | fabesetron |
| 418 | 33667517 | fat cholesterol | 33203 | 56 | alpha1-sitosterol | (Z)-24-ethylidenelophenol |
| 419 | 33667517 | fat cholesterol | 33566 | 56 | a catechol | catechols |
| 420 | 33667517 | fat cholesterol | 34310 | 56 | 24 ( s ) -hydroxycholesterol | (24S)-24-hydroxycholesterol |
| 421 | 33667517 | fat cholesterol | 34610 | 56 | carbestrol | Carbestrol |
| 422 | 33667517 | fat cholesterol | 34671 | 56 | decosteron | Deoxycorticosterone acetate |
| 423 | 33667517 | fat cholesterol | 34896 | 56 | methylestrenolone | Normethandrolone |
| 424 | 33667517 | fat cholesterol | 35515 | 56 | 5alpha-cholestane | 5alpha-cholestane |
| 425 | 33667517 | fat cholesterol | 35517 | 56 | ( 5beta ) -cholestane | 5beta-cholestane |
| 426 | 33667517 | fat cholesterol | 37387 | 56 | calteridol | H3HP-DO3A |
| 427 | 33667517 | fat cholesterol | 41633 | 56 | ( 24s ) -25-epoxycholesterol | 24(S),25-epoxycholesterol |
| 428 | 33667517 | fat cholesterol | 41633 | 56 | 24s,25-epoxy-cholesterol | 24(S),25-epoxycholesterol |
| 429 | 33667517 | fat cholesterol | 42530 | 56 | estosteril | peracetic acid |
| 430 | 33667517 | fat cholesterol | 50516 | 56 | 24 ( r ) -hydroxycholesterol | (24R)-24-hydroxycholesterol |
| 431 | 33667517 | fat cholesterol | 51847 | 56 | keto-ester | ketoester |
| 432 | 33667517 | fat cholesterol | 51847 | 56 | keto ester | ketoester |
| 433 | 33667517 | fat cholesterol | 51847 | 56 | ketoesters | ketoester |
| 434 | 33667517 | fat cholesterol | 52379 | 56 | fecosteryl oleate | fecosteryl oleate |
| 435 | 33667517 | fat cholesterol | 53030 | 56 | oxysterols | oxysterol |
| 436 | 33667517 | fat cholesterol | 60160 | 56 | polyester polymer | polyester polymer |
| 437 | 33667517 | fat cholesterol | 61656 | 56 | cholesteryl glycoside | cholesteryl glycoside |
| 438 | 33667517 | fat cholesterol | 61829 | 56 | 15-ketocholestene | 3beta-hydroxy-5alpha-cholest-8(14)-en-15-one |
| 439 | 33667517 | fat cholesterol | 61835 | 56 | 15-ketocholestane | 3beta-hydroxy-5alpha-cholestan-15-one |
| 440 | 33667517 | fat cholesterol | 63043 | 56 | salt peter | potassium nitrate |
| 441 | 33667517 | fat cholesterol | 63906 | 56 | 5-cholesten-3-one | cholest-5-en-3-one |
| 442 | 33667517 | fat cholesterol | 64762 | 56 | dehydroergosterol | dehydroergosterol |
| 443 | 33667517 | fat cholesterol | 66484 | 56 | silvestrol | silvestrol |
| 444 | 33667517 | fat cholesterol | 66918 | 56 | dihydroergosterol | ergosta-5,7-dien-3beta-ol |
| 445 | 33667517 | fat cholesterol | 67105 | 56 | insect chemosterilant | insect sterilant |
| 446 | 33667517 | fat cholesterol | 67128 | 56 | manchester yellow | 2,4-dinitro-1-naphthol |
| 447 | 33667517 | fat cholesterol | 70337 | 56 | 6-methoxycerevisterol | (22E,24R)-ergosta-7,22-diene-6beta-methoxy-3beta,5alpha-diol |
| 448 | 33667517 | fat cholesterol | 73257 | 56 | ga20 methyl ester | gibberellin A20 methyl ester |
| 449 | 33667517 | fat cholesterol | 73260 | 56 | ga34 methyl ester | gibberellin A34 methyl ester |
| 450 | 33667517 | fat cholesterol | 77700 | 56 | acetic acid methyl ester | methyl acetate |
| 451 | 33667517 | fat cholesterol | 78242 | 56 | 3-cholesteryl acetate | cholesteryl acetate |
| 452 | 33667517 | fat cholesterol | 79817 | 56 | 1-methylestradiol | 1-Methylestra-1,3,5(10)-triene-3,17beta-diol |
| 453 | 33667517 | fat cholesterol | 81136 | 56 | notopterol | Notopterol |
| 454 | 33667517 | fat cholesterol | 82700 | 56 | olodaterol | olodaterol |
| 455 | 33667517 | fat cholesterol | 82751 | 56 | cholesterol arachidonate | cholesteryl arachidonate |
| 456 | 33667517 | fat cholesterol | 83630 | 56 | aetherolea | essential oil |
| 457 | 33667517 | fat cholesterol | 84304 | 56 | cholesteryl myristate | cholesteryl myristate |
| 458 | 33667517 | fat cholesterol | 84323 | 56 | cholesteryl palmitoleate | cholesteryl palmitoleate |
| 459 | 33667517 | fat cholesterol | 87656 | 56 | a hexanoate ester | hexanoate ester |
| 460 | 33667517 | fat cholesterol | 88754 | 56 | cholesterol 1-osbondoate | CE(22:5(4Z,7Z,10Z,13Z,16Z)) |
| 461 | 33667517 | fat cholesterol | 88795 | 56 | 1-eicosenoyl-cholesterol | CE(20:1(11Z)) |
| 462 | 33667517 | fat cholesterol | 89029 | 56 | cholesteryl caprinate | CE(10:0) |
| 463 | 33667517 | fat cholesterol | 89029 | 56 | cholesteryl decanoate | CE(10:0) |
| 464 | 33667517 | fat cholesterol | 89400 | 56 | fucostanol | stigmastanol |
| 465 | 33667517 | fat cholesterol | 89400 | 56 | 24alpha-ethylcholestanol | stigmastanol |
| 466 | 33667517 | fat cholesterol | 89519 | 56 | 5beta-cholestan-3beta-ol | coprostanol |
| 467 | 33667517 | fat cholesterol | 116735 | 56 | amben ethyl ester | benzocaine |
| 468 | 33667517 | fat cholesterol | 131640 | 56 | androstane sterol | androstane sterol |
| 469 | 33667517 | fat cholesterol | 131799 | 56 | hydroxycholesterol ester | hydroxycholesterol ester |
| 470 | 33667517 | fat cholesterol | 133321 | 56 | acetoprole | acetoprole |
| 471 | 33667517 | fat cholesterol | 134793 | 56 | colenormol | oxydibutanol |
| 472 | 33667517 | fat cholesterol | 135030 | 56 | cioteronel | cioteronel |
| 473 | 33667517 | fat cholesterol | 135263 | 56 | dimethylhexestrol | methestrol |
| 474 | 33667517 | fat cholesterol | 135372 | 56 | chlortestosterone | clostebol |
| 475 | 33667517 | fat cholesterol | 135591 | 56 | coumetarol | coumetarol |
| 476 | 33667517 | fat cholesterol | 135834 | 56 | cutisterol | formocortal |
| 477 | 33667517 | fat cholesterol | 135998 | 56 | iodocholesterol ( 131i ) | iodocholesterol (131I) |
| 478 | 33667517 | fat cholesterol | 138126 | 56 | lte4 methyl ester | leukotriene E4 methyl ester |
| 479 | 33667517 | fat cholesterol | 138169 | 56 | desulfated cholecystokinin-8 | Asp-Tyr-Met-Gly-Trp-Met-Asp-Phe-NH2 |
| 480 | 33667517 | fat cholesterol | 138742 | 56 | cholesteryl succinate | cholesteryl hemisuccinate |
| 481 | 33667517 | fat cholesterol | 142077 | 56 | abediterol | abediterol |
| 482 | 33667517 | fat cholesterol | 142773 | 56 | estetrolum | estetrol |
| 483 | 33667517 | fat cholesterol | 149226 | 56 | phenoterol | fenoterol |
| 484 | 33667517 | fat cholesterol | 177022 | 56 | codioester | palmitate phytyl ester |
| 485 | 33667517 | fat cholesterol | 178192 | 56 | avicholate | avicholate |
| 486 | 33667517 | fat cholesterol | 195280 | 56 | artemetero | artemether |
| 487 | 33667517 | fat cholesterol | 3168 | 55 | brassicasterol | brassicasterol |
| 488 | 33667517 | fat cholesterol | 3641 | 55 | chlortrianisestrol | chlorotrianisene |
| 489 | 33667517 | fat cholesterol | 4518 | 55 | dehydrostilbestrol | dienestrol |
| 490 | 33667517 | fat cholesterol | 4591 | 55 | dihydrotachysterol | dihydrotachysterol |
| 491 | 33667517 | fat cholesterol | 8334 | 55 | poriferasterol | Poriferasterol |
| 492 | 33667517 | fat cholesterol | 8403 | 55 | precholecalciferol | previtamin D3 |
| 493 | 33667517 | fat cholesterol | 10112 | 55 | leutrol | zileuton |
| 494 | 33667517 | fat cholesterol | 16175 | 55 | cholest-4-en-3-one | cholest-4-en-3-one |
| 495 | 33667517 | fat cholesterol | 16827 | 55 | corticosterone | corticosterone |
| 496 | 33667517 | fat cholesterol | 17160 | 55 | 17alpha-oestradiol | 17alpha-estradiol |
| 497 | 33667517 | fat cholesterol | 18378 | 55 | 4-methylcholest-7-en-3-ol | 4alpha-methyl-5alpha-cholest-7-en-3beta-ol |
| 498 | 33667517 | fat cholesterol | 18533 | 55 | methylcholestanone | 5alpha-campestan-3-one |
| 499 | 33667517 | fat cholesterol | 21137 | 55 | fe3-s4 cluster | tri-mu-sulfido-mu3-sulfido-triiron |
| 500 | 33667517 | fat cholesterol | 23092 | 55 | chemosterilant | chemosterilant |
| 501 | 33667517 | fat cholesterol | 23396 | 55 | cortisol ester | cortisol ester |
| 502 | 33667517 | fat cholesterol | 25223 | 55 | mesylate ester | methanesulfonate ester |
| 503 | 33667517 | fat cholesterol | 26981 | 55 | threo-tetritol | threitol |
| 504 | 33667517 | fat cholesterol | 27436 | 55 | methyltestosterone | methyltestosterone |
| 505 | 33667517 | fat cholesterol | 27750 | 55 | acetic acid , ethyl ester | ethyl acetate |
| 506 | 33667517 | fat cholesterol | 27974 | 55 | estriol | estriol |
| 507 | 33667517 | fat cholesterol | 28940 | 55 | colecalciferol | calciol |
| 508 | 33667517 | fat cholesterol | 28955 | 55 | 2-methoxyestradiol | 2-methoxy-17beta-estradiol |
| 509 | 33667517 | fat cholesterol | 29747 | 55 | cholate | cholate |
| 510 | 33667517 | fat cholesterol | 31527 | 55 | hydrogesterone | dydrogesterone |
| 511 | 33667517 | fat cholesterol | 31590 | 55 | falecalcitriol | Falecalcitriol |
| 512 | 33667517 | fat cholesterol | 32019 | 55 | enterol | piromidic acid |
| 513 | 33667517 | fat cholesterol | 33731 | 55 | cluster | cluster |
| 514 | 33667517 | fat cholesterol | 33739 | 55 | 2fe-2s cluster | di-mu-sulfido-diiron(0) |
| 515 | 33667517 | fat cholesterol | 35347 | 55 | 3alpha-sterols | 3alpha-sterol |
| 516 | 33667517 | fat cholesterol | 35559 | 55 | tetrole | furan |
| 517 | 33667517 | fat cholesterol | 35821 | 55 | cholesterol-lowering drug | anticholesteremic drug |
| 518 | 33667517 | fat cholesterol | 36054 | 55 | benzoate ester | benzoate ester |
| 519 | 33667517 | fat cholesterol | 37088 | 55 | anthrol | anthrol |
| 520 | 33667517 | fat cholesterol | 37733 | 55 | anticholineesterase inhibitor | EC 3.1.1.8 (cholinesterase) inhibitor |
| 521 | 33667517 | fat cholesterol | 38069 | 55 | fortrol | cyanazine |
| 522 | 33667517 | fat cholesterol | 38083 | 55 | malonate ester | malonate ester |
| 523 | 33667517 | fat cholesterol | 38462 | 55 | true cholinesterase inhibitor | EC 3.1.1.7 (acetylcholinesterase) inhibitor |
| 524 | 33667517 | fat cholesterol | 41633 | 55 | 24 ( s ) ,25-epoxycholesterol | 24(S),25-epoxycholesterol |
| 525 | 33667517 | fat cholesterol | 41922 | 55 | diethylstilbestrol | diethylstilbestrol |
| 526 | 33667517 | fat cholesterol | 41922 | 55 | trans-diethylstilbesterol | diethylstilbestrol |
| 527 | 33667517 | fat cholesterol | 41922 | 55 | dietilestilbestrol | diethylstilbestrol |
| 528 | 33667517 | fat cholesterol | 42638 | 55 | achless | flufenamic acid |
| 529 | 33667517 | fat cholesterol | 46916 | 55 | acetic acid ethenyl ester | vinyl acetate |
| 530 | 33667517 | fat cholesterol | 47402 | 55 | 3fe-4s cluster | tri-mu-sulfido-mu3-sulfido-triiron(0) |
| 531 | 33667517 | fat cholesterol | 47788 | 55 | a 3-oxosteroid | 3-oxo steroid |
| 532 | 33667517 | fat cholesterol | 47789 | 55 | a 7-oxosteroid | 7-oxo steroid |
| 533 | 33667517 | fat cholesterol | 47806 | 55 | oxocholecalciferol | oxocalciol |
| 534 | 33667517 | fat cholesterol | 49305 | 55 | cholesterol-alpha-epoxide | 5,6alpha-epoxy-5alpha-cholestan-3beta-ol |
| 535 | 33667517 | fat cholesterol | 50424 | 55 | acrylate ester | acrylate ester |
| 536 | 33667517 | fat cholesterol | 50477 | 55 | butyrate ester | butyrate ester |
| 537 | 33667517 | fat cholesterol | 50751 | 55 | anti-oestrogen | anti-estrogen |
| 538 | 33667517 | fat cholesterol | 50784 | 55 | pivalate ester | pivalate ester |
| 539 | 33667517 | fat cholesterol | 50858 | 55 | corticosteroid | corticosteroid |
| 540 | 33667517 | fat cholesterol | 50871 | 55 | valerate ester | valerate ester |
| 541 | 33667517 | fat cholesterol | 50979 | 55 | boronate ester | boronate ester |
| 542 | 33667517 | fat cholesterol | 51784 | 55 | atto 425 nhs-ester | ATTO 425-3 |
| 543 | 33667517 | fat cholesterol | 51789 | 55 | atto 465 nhs-ester | ATTO 465-3 |
| 544 | 33667517 | fat cholesterol | 51806 | 55 | atto 495 nhs-ester | ATTO 495-3 |
| 545 | 33667517 | fat cholesterol | 51813 | 55 | atto 520 nhs-ester | ATTO 520-3 |
| 546 | 33667517 | fat cholesterol | 51826 | 55 | atto 610 nhs-ester | ATTO 610-3 |
| 547 | 33667517 | fat cholesterol | 51830 | 55 | atto 635 nhs-ester | ATTO 635-3 |
| 548 | 33667517 | fat cholesterol | 51849 | 55 | beta-ketoester | beta-ketoester |
| 549 | 33667517 | fat cholesterol | 52393 | 55 | an episterol ester | episterol ester |
| 550 | 33667517 | fat cholesterol | 52615 | 55 | 4-formylzymosterol | 4-formylzymosterol |
| 551 | 33667517 | fat cholesterol | 52972 | 55 | 5-dehydroepisterol | ergosta-5,7,24(28)-trien-3beta-ol |
| 552 | 33667517 | fat cholesterol | 59567 | 55 | ( r ) -clenbuterol | (R)-clenbuterol |
| 553 | 33667517 | fat cholesterol | 59567 | 55 | ( - ) -clenbuterol | (R)-clenbuterol |
| 554 | 33667517 | fat cholesterol | 59568 | 55 | ( s ) -clenbuterol | (S)-clenbuterol |
| 555 | 33667517 | fat cholesterol | 59568 | 55 | ( + ) -clenbuterol | (S)-clenbuterol |
| 556 | 33667517 | fat cholesterol | 59602 | 55 | colestipol hcl | colestipol hydrochloride |
| 557 | 33667517 | fat cholesterol | 60935 | 55 | sinapate ester | sinapate ester |
| 558 | 33667517 | fat cholesterol | 62732 | 55 | aromatic ester | aromatic ester |
| 559 | 33667517 | fat cholesterol | 63841 | 55 | 4-methylzymosterol | 4-methylzymosterol |
| 560 | 33667517 | fat cholesterol | 63906 | 55 | cholest-5-en-3-one | cholest-5-en-3-one |
| 561 | 33667517 | fat cholesterol | 63906 | 55 | delta ( 5 ) -cholestenone | cholest-5-en-3-one |
| 562 | 33667517 | fat cholesterol | 64198 | 55 | dithioglycerol | dimercaprol |
| 563 | 33667517 | fat cholesterol | 64426 | 55 | cyclitol ester | cyclitol ester |
| 564 | 33667517 | fat cholesterol | 67593 | 55 | sitostanetriol | stigmastane-3beta,5alpha,6beta-triol |
| 565 | 33667517 | fat cholesterol | 68597 | 55 | clathsterol ( 2- ) | clathsterol(2-) |
| 566 | 33667517 | fat cholesterol | 69241 | 55 | totarol | totarol |
| 567 | 33667517 | fat cholesterol | 70609 | 55 | feselol | feselol |
| 568 | 33667517 | fat cholesterol | 74253 | 55 | mycolate ester | mycolate ester |
| 569 | 33667517 | fat cholesterol | 74537 | 55 | 1-thioglycerol | monothioglycerol |
| 570 | 33667517 | fat cholesterol | 75925 | 55 | stearate ester | octadecanoate ester |
| 571 | 33667517 | fat cholesterol | 77175 | 55 | chlorinate steroid | chlorinated steroid |
| 572 | 33667517 | fat cholesterol | 77995 | 55 | bathophenanthrolin | 4,7-diphenyl-1,10-phenanthroline |
| 573 | 33667517 | fat cholesterol | 78242 | 55 | cholesterol 3beta-acetate | cholesteryl acetate |
| 574 | 33667517 | fat cholesterol | 83351 | 55 | tosylate ester | tosylate ester |
| 575 | 33667517 | fat cholesterol | 84323 | 55 | 16:1 ( 9z ) cholesterol ester | cholesteryl palmitoleate |
| 576 | 33667517 | fat cholesterol | 84576 | 55 | ( 25s ) -cholestenoyl-coa | (25S)-3beta-hydroxy-5-cholesten-26-oyl-CoA |
| 577 | 33667517 | fat cholesterol | 84932 | 55 | palmitic acid ethyl ester | ethyl hexadecanoate |
| 578 | 33667517 | fat cholesterol | 86315 | 55 | a methyl thioether | methyl sulfide |
| 579 | 33667517 | fat cholesterol | 86618 | 55 | enanthic acid ethyl ester | ethyl heptanoate |
| 580 | 33667517 | fat cholesterol | 87051 | 55 | methost-8-enol | 4alpha-methyl-5alpha-cholest-8-en-3beta-ol |
| 581 | 33667517 | fat cholesterol | 87620 | 55 | actriol | 16beta-hydroxyestradiol |
| 582 | 33667517 | fat cholesterol | 87656 | 55 | caproate ester | hexanoate ester |
| 583 | 33667517 | fat cholesterol | 87657 | 55 | an octanoate ester | octanoate ester |
| 584 | 33667517 | fat cholesterol | 88740 | 55 | 1-lignoceroyl-cholesterol | CE(24:0) |
| 585 | 33667517 | fat cholesterol | 88758 | 55 | 14:1 ( 9z ) cholesterol ester | CE(14:1(9Z)) |
| 586 | 33667517 | fat cholesterol | 88763 | 55 | cholesterol 1-arachidonyl | CE(20:0) |
| 587 | 33667517 | fat cholesterol | 88763 | 55 | cholesterol 1-eicosanoate | CE(20:0) |
| 588 | 33667517 | fat cholesterol | 88763 | 55 | cholesteryl 1-arachidonyl | CE(20:0) |
| 589 | 33667517 | fat cholesterol | 88795 | 55 | cholesterol 1-eicosenoate | CE(20:1(11Z)) |
| 590 | 33667517 | fat cholesterol | 89029 | 55 | cholesterol decanoic acid | CE(10:0) |
| 591 | 33667517 | fat cholesterol | 89760 | 55 | solerol | Solerol |
| 592 | 33667517 | fat cholesterol | 89886 | 55 | homocatechol methyl ester | 2-Methoxy-4-methylphenol |
| 593 | 33667517 | fat cholesterol | 90320 | 55 | pyruvate ester | pyruvate ester |
| 594 | 33667517 | fat cholesterol | 131638 | 55 | cycloartane sterol | cycloartane sterol |
| 595 | 33667517 | fat cholesterol | 131641 | 55 | estrane sterol | estrane sterol |
| 596 | 33667517 | fat cholesterol | 131643 | 55 | oxocholestanes | ketocholestane |
| 597 | 33667517 | fat cholesterol | 131703 | 55 | stigmastane sterol | stigmastane sterol |
| 598 | 33667517 | fat cholesterol | 134857 | 55 | ethylarterenol | ethylnorepinephrine |
| 599 | 33667517 | fat cholesterol | 135983 | 55 | forsteo | teriparatide |
| 600 | 33667517 | fat cholesterol | 136975 | 55 | 4-methoxyestradiol | 4-methoxy-17beta-estradiol |
| 601 | 33667517 | fat cholesterol | 139463 | 55 | farnesiferol a | Farnesiferol A |
| 602 | 33667517 | fat cholesterol | 145561 | 55 | macrotetrolide | macrotetrolide |
| 603 | 33667517 | fat cholesterol | 172781 | 55 | enanthic acid hexyl ester | hexyl heptanoate |
| 604 | 33667517 | fat cholesterol | 1294 | 54 | 20,22-dihydroxycholesterol | (20R,22R)-20,22-dihydroxycholesterol |
| 605 | 33667517 | fat cholesterol | 2440 | 54 | acrovestone | acrovestone |
| 606 | 33667517 | fat cholesterol | 5686 | 54 | heterocycle | heterocyclic compound |
| 607 | 33667517 | fat cholesterol | 6469 | 54 | acetic acid linalool ester | linalyl acetate |
| 608 | 33667517 | fat cholesterol | 8082 | 54 | acetic acid , phenyl ester | phenyl acetate |
| 609 | 33667517 | fat cholesterol | 8391 | 54 | prenalterol | Prenalterol |
| 610 | 33667517 | fat cholesterol | 9536 | 54 | thioseconal | thiamylal |
| 611 | 33667517 | fat cholesterol | 9622 | 54 | tolterodine | tolterodine |
| 612 | 33667517 | fat cholesterol | 9622 | 54 | tolterodina | tolterodine |
| 613 | 33667517 | fat cholesterol | 10036 | 54 | a wax ester | wax ester |
| 614 | 33667517 | fat cholesterol | 10333 | 54 | spinasterol | alpha-Spinasterol |
| 615 | 33667517 | fat cholesterol | 15709 | 54 | xanthotoxol | xanthotoxol |
| 616 | 33667517 | fat cholesterol | 16514 | 54 | chrysoeriol | 4',5,7-trihydroxy-3'-methoxyflavone |
| 617 | 33667517 | fat cholesterol | 17026 | 54 | progesteron | progesterone |
| 618 | 33667517 | fat cholesterol | 17347 | 54 | testosteron | testosterone |
| 619 | 33667517 | fat cholesterol | 17737 | 54 | desmosterol | desmosterol |
| 620 | 33667517 | fat cholesterol | 27013 | 54 | tocoferoles | tocopherol |
| 621 | 33667517 | fat cholesterol | 27373 | 54 | pantothenol | pantothenol |
| 622 | 33667517 | fat cholesterol | 27584 | 54 | aldosterone | aldosterone |
| 623 | 33667517 | fat cholesterol | 27693 | 54 | nimbosterol | sitosterol |
| 624 | 33667517 | fat cholesterol | 27957 | 54 | methylketol | hydroxyacetone |
| 625 | 33667517 | fat cholesterol | 28054 | 54 | orthocresol | o-cresol |
| 626 | 33667517 | fat cholesterol | 31428 | 54 | colestimide | Colestilan |
| 627 | 33667517 | fat cholesterol | 31643 | 54 | gadoteridol | gadoteridol |
| 628 | 33667517 | fat cholesterol | 31790 | 54 | lynestrenol | Lynestrenol |
| 629 | 33667517 | fat cholesterol | 31790 | 54 | linestrenol | Lynestrenol |
| 630 | 33667517 | fat cholesterol | 33235 | 54 | tocotrienol | tocotrienol |
| 631 | 33667517 | fat cholesterol | 34583 | 54 | bolasterone | Bolasterone |
| 632 | 33667517 | fat cholesterol | 34671 | 54 | decosterone | Deoxycorticosterone acetate |
| 633 | 33667517 | fat cholesterol | 34749 | 54 | ethisterone | ethisterone |
| 634 | 33667517 | fat cholesterol | 34798 | 54 | indanestrol | Indanestrol |
| 635 | 33667517 | fat cholesterol | 34903 | 54 | oxymesteron | Oranabol |
| 636 | 33667517 | fat cholesterol | 35515 | 54 | ( 5alpha ) -cholestane | 5alpha-cholestane |
| 637 | 33667517 | fat cholesterol | 35702 | 54 | ethyl ether | diethyl ether |
| 638 | 33667517 | fat cholesterol | 35789 | 54 | ketosteroid | oxo steroid |
| 639 | 33667517 | fat cholesterol | 35821 | 54 | cholesterol-lowering agent | anticholesteremic drug |
| 640 | 33667517 | fat cholesterol | 37864 | 54 | thioperoxol | thioperoxol |
| 641 | 33667517 | fat cholesterol | 40933 | 54 | androstenol | 5alpha-androst-16-en-3alpha-ol |
| 642 | 33667517 | fat cholesterol | 41922 | 54 | trans-diethylstilboesterol | diethylstilbestrol |
| 643 | 33667517 | fat cholesterol | 47799 | 54 | calcitetrol | calcitetrol |
| 644 | 33667517 | fat cholesterol | 51305 | 54 | acene ester | acene ester |
| 645 | 33667517 | fat cholesterol | 52450 | 54 | paf-acether | 2-O-acetyl-1-O-octadecyl-sn-glycero-3-phosphocholine |
| 646 | 33667517 | fat cholesterol | 52450 | 54 | paf acether | 2-O-acetyl-1-O-octadecyl-sn-glycero-3-phosphocholine |
| 647 | 33667517 | fat cholesterol | 53767 | 54 | chlorseptol | chloramine T |
| 648 | 33667517 | fat cholesterol | 59211 | 54 | cefotiam hexetil ester | cefotiam hexetil ester |
| 649 | 33667517 | fat cholesterol | 59594 | 54 | colesevelam | colesevelam |
| 650 | 33667517 | fat cholesterol | 60523 | 54 | a phosphatidylglycerol | phosphatidylglycerol(1-) |
| 651 | 33667517 | fat cholesterol | 62452 | 54 | achilleol c | camelliol C |
| 652 | 33667517 | fat cholesterol | 62874 | 54 | globosterol | globosterol |
| 653 | 33667517 | fat cholesterol | 63461 | 54 | achilleol b | achilleol B |
| 654 | 33667517 | fat cholesterol | 64983 | 54 | alternariol | alternariol |
| 655 | 33667517 | fat cholesterol | 65368 | 54 | aculeatol e | aculeatol E |
| 656 | 33667517 | fat cholesterol | 67105 | 54 | insect chemosterilants | insect sterilant |
| 657 | 33667517 | fat cholesterol | 67554 | 54 | daucosterin | daucosterol |
| 658 | 33667517 | fat cholesterol | 68575 | 54 | indacaterol | indacaterol |
| 659 | 33667517 | fat cholesterol | 69432 | 54 | fungisterol | fungisterol |
| 660 | 33667517 | fat cholesterol | 69435 | 54 | 4,4-dimethylfecosterol | 4,4-dimethyl-5alpha-ergosta-8,24(28)-dien-3beta-ol |
| 661 | 33667517 | fat cholesterol | 69662 | 54 | athrolide e | Athrolide E |
| 662 | 33667517 | fat cholesterol | 70337 | 54 | 6-o-methylcerevisterol | (22E,24R)-ergosta-7,22-diene-6beta-methoxy-3beta,5alpha-diol |
| 663 | 33667517 | fat cholesterol | 70869 | 54 | hippurate methyl ester | methyl hippurate |
| 664 | 33667517 | fat cholesterol | 71567 | 54 | ( 25s ) -cholestenoate | (25S)-cholestenoate |
| 665 | 33667517 | fat cholesterol | 73641 | 54 | lactic acid , methyl ester | rac-methyl lactate |
| 666 | 33667517 | fat cholesterol | 74103 | 54 | cholesterol ester ( 22:6 ) | CE(22:6) |
| 667 | 33667517 | fat cholesterol | 74611 | 54 | d-lactate methyl ester | methyl (R)-lactate |
| 668 | 33667517 | fat cholesterol | 74611 | 54 | d-lactic acid methyl ester | methyl (R)-lactate |
| 669 | 33667517 | fat cholesterol | 75631 | 54 | cetyl alcohol stearate | palmityl stearate |
| 670 | 33667517 | fat cholesterol | 77006 | 54 | ovasterol-b | 17beta-estradiol 3-benzoate |
| 671 | 33667517 | fat cholesterol | 78242 | 54 | ( - ) -cholesteryl acetate | cholesteryl acetate |
| 672 | 33667517 | fat cholesterol | 81252 | 54 | bitocholate | Bitocholate |
| 673 | 33667517 | fat cholesterol | 81787 | 54 | cafenstrole | cafenstrole |
| 674 | 33667517 | fat cholesterol | 83222 | 54 | l-lactate methyl ester | methyl (S)-lactate |
| 675 | 33667517 | fat cholesterol | 83222 | 54 | l-lactic acid methyl ester | methyl (S)-lactate |
| 676 | 33667517 | fat cholesterol | 84304 | 54 | cholesterol ester ( 14:0 ) | cholesteryl myristate |
| 677 | 33667517 | fat cholesterol | 86145 | 54 | cholest-5-ene-3,7,22-triol | (7alpha,22R)-dihydroxycholesterol |
| 678 | 33667517 | fat cholesterol | 87388 | 54 | fumaric acid diethyl ester | diethyl fumarate |
| 679 | 33667517 | fat cholesterol | 88258 | 54 | sulfametrol | sulfametrole |
| 680 | 33667517 | fat cholesterol | 88740 | 54 | cholesterol ester ( 24:0 ) | CE(24:0) |
| 681 | 33667517 | fat cholesterol | 88740 | 54 | cholesterol 1-lignoceroate | CE(24:0) |
| 682 | 33667517 | fat cholesterol | 88753 | 54 | cholesterol 1-erucoic acid | CE(22:1(13Z)) |
| 683 | 33667517 | fat cholesterol | 88753 | 54 | cholesterol ester ( 22:1 ) | CE(22:1(13Z)) |
| 684 | 33667517 | fat cholesterol | 88753 | 54 | cholesteryl 1-erucoate | CE(22:1(13Z)) |
| 685 | 33667517 | fat cholesterol | 88754 | 54 | cholesterol ester ( 22:5 ) | CE(22:5(4Z,7Z,10Z,13Z,16Z)) |
| 686 | 33667517 | fat cholesterol | 88755 | 54 | cholesterol 1-eicsoic acid | CE(20:4(8Z,11Z,14Z,17Z)) |
| 687 | 33667517 | fat cholesterol | 88755 | 54 | cholesterol ester ( 20:4 ) | CE(20:4(8Z,11Z,14Z,17Z)) |
| 688 | 33667517 | fat cholesterol | 88755 | 54 | cholesteryl 1-eicsoate | CE(20:4(8Z,11Z,14Z,17Z)) |
| 689 | 33667517 | fat cholesterol | 88758 | 54 | cholesterol ester ( 14:1 ) | CE(14:1(9Z)) |
| 690 | 33667517 | fat cholesterol | 88763 | 54 | cholesteryl arachidate | CE(20:0) |
| 691 | 33667517 | fat cholesterol | 88763 | 54 | cholesterol ester ( 20:0 ) | CE(20:0) |
| 692 | 33667517 | fat cholesterol | 88768 | 54 | ( 11z-octadecenoyl ) -cholesterol | (11Z-octadecenoyl)-cholesterol |
| 693 | 33667517 | fat cholesterol | 88795 | 54 | cholesterol ester ( 20:1 ) | CE(20:1(11Z)) |
| 694 | 33667517 | fat cholesterol | 89029 | 54 | cholesteryl n-decylate | CE(10:0) |
| 695 | 33667517 | fat cholesterol | 131652 | 54 | cholesteryl-coa ( 4- ) | cholestanoyl-CoA(4-) |
| 696 | 33667517 | fat cholesterol | 132824 | 54 | spathulenol | spathulenol |
| 697 | 33667517 | fat cholesterol | 135264 | 54 | benzoestrol | benzestrol |
| 698 | 33667517 | fat cholesterol | 135293 | 54 | mesterolone | mesterolone |
| 699 | 33667517 | fat cholesterol | 135356 | 54 | calusterone | calusterone |
| 700 | 33667517 | fat cholesterol | 135437 | 54 | cefacetrile | cefacetrile |
| 701 | 33667517 | fat cholesterol | 135567 | 54 | penmesterol | penmesterol |
| 702 | 33667517 | fat cholesterol | 135993 | 54 | nomegestrol | nomegestrol |
| 703 | 33667517 | fat cholesterol | 136579 | 54 | cholesterol sulfate ( 1- ) | cholesterol sulfate(1-) |
| 704 | 33667517 | fat cholesterol | 137552 | 54 | cholesterol ester ( 16:1 ) | CE(16:1) |
| 705 | 33667517 | fat cholesterol | 137554 | 54 | cholesterol ester ( 17:1 ) | CE(17:1) |
| 706 | 33667517 | fat cholesterol | 137555 | 54 | cholesterol ester ( 17:2 ) | CE(17:2) |
| 707 | 33667517 | fat cholesterol | 137556 | 54 | cholesterol ester ( 19:2 ) | CE(19:2) |
| 708 | 33667517 | fat cholesterol | 137557 | 54 | cholesterol ester ( 19:3 ) | CE(19:3) |
| 709 | 33667517 | fat cholesterol | 137661 | 54 | allocholate | allocholate |
| 710 | 33667517 | fat cholesterol | 140435 | 54 | cholesterol-2,2,3,4,4,6-d6 | cholesterol-2,2,3,4,4,6-d6 |
| 711 | 33667517 | fat cholesterol | 144075 | 54 | arthothelin | arthothelin |
| 712 | 33667517 | fat cholesterol | 149226 | 54 | fenoterolum | fenoterol |
| 713 | 33667517 | fat cholesterol | 155905 | 54 | pincasterol | crinosterol |
| 714 | 33667517 | fat cholesterol | 177022 | 54 | palmitate phytyl ester | palmitate phytyl ester |
| 715 | 33667517 | fat cholesterol | 177026 | 54 | myristate phytyl ester | myristate phytyl ester |
| 716 | 33667517 | fat cholesterol | 177028 | 54 | decanoate phytyl ester | caprate phytyl ester |
| 717 | 33667517 | fat cholesterol | 1296 | 53 | ( 20s ) -20-hydroxycholesterol | 20-hydroxycholesterol |
| 718 | 33667517 | fat cholesterol | 1301 | 53 | ( 22s ) -22-hydroxycholesterol | (22S)-22-hydroxycholesterol |
| 719 | 33667517 | fat cholesterol | 1949 | 53 | 4alpha-methylzymosterol | 4alpha-methylzymosterol |
| 720 | 33667517 | fat cholesterol | 3641 | 53 | chloortrianisestrol | chlorotrianisene |
| 721 | 33667517 | fat cholesterol | 4489 | 53 | thioallyl ether | Diallyl sulfide |
| 722 | 33667517 | fat cholesterol | 4777 | 53 | dihydrolysergol | Elymoclavine |
| 723 | 33667517 | fat cholesterol | 4867 | 53 | methyl chavicole ( estragole ) | Estragole |
| 724 | 33667517 | fat cholesterol | 4903 | 53 | 17-ethinylestradiol | 17alpha-ethynylestradiol |
| 725 | 33667517 | fat cholesterol | 5120 | 53 | fluoxymesterone | fluoxymesterone |
| 726 | 33667517 | fat cholesterol | 5120 | 53 | fluoximesterona | fluoxymesterone |
| 727 | 33667517 | fat cholesterol | 6808 | 53 | methallenestril | Methallenestril |
| 728 | 33667517 | fat cholesterol | 7794 | 53 | phosphate monoester | phosphate monoester |
| 729 | 33667517 | fat cholesterol | 16236 | 53 | alcohol etilico | ethanol |
| 730 | 33667517 | fat cholesterol | 16290 | 53 | cholesta-7,24-dien-3-ol | 5alpha-cholesta-7,24-dien-3beta-ol |
| 731 | 33667517 | fat cholesterol | 16822 | 53 | choline sulfate | choline sulfate |
| 732 | 33667517 | fat cholesterol | 17160 | 53 | alpha-estradiol | 17alpha-estradiol |
| 733 | 33667517 | fat cholesterol | 18431 | 53 | 7alpha,26-dihydroxycholesterol | 7alpha,26-dihydroxycholesterol |
| 734 | 33667517 | fat cholesterol | 20651 | 53 | 5-dihydroergosterol | 5alpha-ergosta-7,22-dien-3beta-ol |
| 735 | 33667517 | fat cholesterol | 21415 | 53 | l-tyrosyl ester | L-tyrosyl ester |
| 736 | 33667517 | fat cholesterol | 22327 | 53 | aliphatic thioether | aliphatic sulfide |
| 737 | 33667517 | fat cholesterol | 23003 | 53 | carbamate ester | carbamate ester |
| 738 | 33667517 | fat cholesterol | 23092 | 53 | chemosterilants | chemosterilant |
| 739 | 33667517 | fat cholesterol | 23203 | 53 | cholestenoyl-coenzyme a | cholestenoyl-CoA |
| 740 | 33667517 | fat cholesterol | 24532 | 53 | organic heterocycle | organic heterocyclic compound |
| 741 | 33667517 | fat cholesterol | 25835 | 53 | palmitate ester | hexadecanoate ester |
| 742 | 33667517 | fat cholesterol | 25835 | 53 | palmitic acid ester | hexadecanoate ester |
| 743 | 33667517 | fat cholesterol | 27436 | 53 | methyltestosteronum | methyltestosterone |
| 744 | 33667517 | fat cholesterol | 27710 | 53 | ethyl thioether | diethyl sulfide |
| 745 | 33667517 | fat cholesterol | 27710 | 53 | thioethyl ether | diethyl sulfide |
| 746 | 33667517 | fat cholesterol | 28082 | 53 | 3beta,5alpha,6beta-cholestanetriol | 5alpha-cholestane-3beta,5,6beta-triol |
| 747 | 33667517 | fat cholesterol | 28575 | 53 | l-glutamate methylester | L-glutamate methyl ester |
| 748 | 33667517 | fat cholesterol | 28604 | 53 | ( 24z ) -ethylidenecholesterol | isofucosterol |
| 749 | 33667517 | fat cholesterol | 31221 | 53 | anetholtrithion | Anetholtrithion |
| 750 | 33667517 | fat cholesterol | 31410 | 53 | clenbuterol hcl | clenbuterol hydrochloride |
| 751 | 33667517 | fat cholesterol | 31590 | 53 | falecalcitoriol | Falecalcitriol |
| 752 | 33667517 | fat cholesterol | 34179 | 53 | 17alpha-methylestradiol | 17alpha-Methylestradiol |
| 753 | 33667517 | fat cholesterol | 34310 | 53 | ( 24s ) -24-hydroxycholesterol | (24S)-24-hydroxycholesterol |
| 754 | 33667517 | fat cholesterol | 34310 | 53 | cholest-5-ene-3,24-diol | (24S)-24-hydroxycholesterol |
| 755 | 33667517 | fat cholesterol | 34717 | 53 | dimethylstilbestrol | Dimethylstilbestrol |
| 756 | 33667517 | fat cholesterol | 34749 | 53 | ethynyltestosterone | ethisterone |
| 757 | 33667517 | fat cholesterol | 35484 | 53 | phthalate ester | phthalate ester |
| 758 | 33667517 | fat cholesterol | 35866 | 53 | 5beta-cholest-7-ene | 5beta-cholest-7-ene |
| 759 | 33667517 | fat cholesterol | 36087 | 53 | cinnamate ester | cinnamate ester |
| 760 | 33667517 | fat cholesterol | 36181 | 53 | succinate ester | succinate ester |
| 761 | 33667517 | fat cholesterol | 36818 | 53 | seco-cholestane | seco-cholestane |
| 762 | 33667517 | fat cholesterol | 37505 | 53 | anthracenetriol | anthracenetriol |
| 763 | 33667517 | fat cholesterol | 37623 | 53 | 7alpha,25-dihydroxycholesterol | 7alpha,25-dihydroxycholesterol |
| 764 | 33667517 | fat cholesterol | 38133 | 53 | naphthalenediol | naphthalenediol |
| 765 | 33667517 | fat cholesterol | 38462 | 53 | acetylcholinesterase inhibitor | EC 3.1.1.7 (acetylcholinesterase) inhibitor |
| 766 | 33667517 | fat cholesterol | 42156 | 53 | 17alpha-estriol | 17-epiestriol |
| 767 | 33667517 | fat cholesterol | 46722 | 53 | carbonate ester | carbonate ester |
| 768 | 33667517 | fat cholesterol | 46916 | 53 | acetic acid vinyl ester | vinyl acetate |
| 769 | 33667517 | fat cholesterol | 47739 | 53 | nife4s4 cluster | NiFe4S4 cluster |
| 770 | 33667517 | fat cholesterol | 47771 | 53 | beta-tocopherol | beta-tocopherol |
| 771 | 33667517 | fat cholesterol | 47786 | 53 | a 16-oxosteroid | 16-oxo steroid |
| 772 | 33667517 | fat cholesterol | 47806 | 53 | oxocholecalciferols | oxocalciol |
| 773 | 33667517 | fat cholesterol | 48619 | 53 | thioglycolate ester | thioglycolate ester |
| 774 | 33667517 | fat cholesterol | 48796 | 53 | fe7mos9 cluster | iron-sulfur-molybdenum cluster |
| 775 | 33667517 | fat cholesterol | 48796 | 53 | fe-mo-s cluster | iron-sulfur-molybdenum cluster |
| 776 | 33667517 | fat cholesterol | 48873 | 53 | anticholinergic | cholinergic antagonist |
| 777 | 33667517 | fat cholesterol | 50477 | 53 | butanoate ester | butyrate ester |
| 778 | 33667517 | fat cholesterol | 50516 | 53 | ( 24r ) -24-hydroxycholesterol | (24R)-24-hydroxycholesterol |
| 779 | 33667517 | fat cholesterol | 50517 | 53 | 7alpha,24-dihydroxycholesterol | 7alpha,24-dihydroxycholesterol |
| 780 | 33667517 | fat cholesterol | 50830 | 53 | fluorinated steroid | fluorinated steroid |
| 781 | 33667517 | fat cholesterol | 50850 | 53 | etabonate ester | etabonate ester |
| 782 | 33667517 | fat cholesterol | 50852 | 53 | glycinate ester | glycinyl ester |
| 783 | 33667517 | fat cholesterol | 50898 | 53 | enanthate ester | heptanoate ester |
| 784 | 33667517 | fat cholesterol | 51848 | 53 | alpha-ketoester | alpha-ketoester |
| 785 | 33667517 | fat cholesterol | 51849 | 53 | beta-keto-ester | beta-ketoester |
| 786 | 33667517 | fat cholesterol | 51849 | 53 | beta-keto ester | beta-ketoester |
| 787 | 33667517 | fat cholesterol | 51849 | 53 | beta-ketoesters | beta-ketoester |
| 788 | 33667517 | fat cholesterol | 52393 | 53 | episterol ester | episterol ester |
| 789 | 33667517 | fat cholesterol | 52464 | 53 | jasmonate ester | jasmonate ester |
| 790 | 33667517 | fat cholesterol | 53519 | 53 | graft copolymer | graft copolymer |
| 791 | 33667517 | fat cholesterol | 59114 | 53 | catechol dimethyl ether | veratrole |
| 792 | 33667517 | fat cholesterol | 60400 | 53 | ni/fe/s cluster | nickel-iron-sulfur cluster |
| 793 | 33667517 | fat cholesterol | 61164 | 53 | l-alanate ester | L-alanyl ester |
| 794 | 33667517 | fat cholesterol | 61165 | 53 | d-alanate ester | D-alanyl ester |
| 795 | 33667517 | fat cholesterol | 61258 | 53 | nife3s4 cluster | NiFe3S4 cluster |
| 796 | 33667517 | fat cholesterol | 63005 | 53 | chile saltpeter | sodium nitrate |
| 797 | 33667517 | fat cholesterol | 63640 | 53 | 3-oxocholest-4-en-26-ol | 26-hydroxycholest-4-en-3-one |
| 798 | 33667517 | fat cholesterol | 64292 | 53 | coniferyl ester | coniferyl ester |
| 799 | 33667517 | fat cholesterol | 67237 | 53 | ( 22r ) -22-hydroxycholesterol | (22R)-22-hydroxycholesterol |
| 800 | 33667517 | fat cholesterol | 68597 | 53 | clathsterol dianion | clathsterol(2-) |
| 801 | 33667517 | fat cholesterol | 74537 | 53 | alpha-thiolglycerol | monothioglycerol |
| 802 | 33667517 | fat cholesterol | 75622 | 53 | palmityl oleate | palmityl oleate |
| 803 | 33667517 | fat cholesterol | 76591 | 53 | ( 25r ) -26-hydroxycholesterol | (25R)-cholest-5-ene-3beta,26-diol |
| 804 | 33667517 | fat cholesterol | 77995 | 53 | bathophenanthroline | 4,7-diphenyl-1,10-phenanthroline |
| 805 | 33667517 | fat cholesterol | 79825 | 53 | 1-methylestrone | 3-Hydroxy-1-methylestra-1,3,5(10)-trien-17-one |
| 806 | 33667517 | fat cholesterol | 81216 | 53 | chrysanthetriol | Chrysanthetriol |
| 807 | 33667517 | fat cholesterol | 81305 | 53 | 7-oxateasterone | 7-Oxateasterone |
| 808 | 33667517 | fat cholesterol | 82750 | 53 | cholestryl stearate | cholesteryl stearate |
| 809 | 33667517 | fat cholesterol | 83631 | 53 | gallic acid octyl ester | octyl gallate |
| 810 | 33667517 | fat cholesterol | 84352 | 53 | cholesteryl docosanoate | cholesteryl behenate |
| 811 | 33667517 | fat cholesterol | 84888 | 53 | ethyl cetoleate | ethyl (11Z)-docosenoate |
| 812 | 33667517 | fat cholesterol | 86087 | 53 | 4beta,24s-dihydroxycholesterol | 4beta,24S-dihydroxycholesterol |
| 813 | 33667517 | fat cholesterol | 86570 | 53 | ( 5alpha ) -cholestan-3beta-ol | (5alpha)-cholestan-3beta-ol |
| 814 | 33667517 | fat cholesterol | 87106 | 53 | fast fuchsine g | acid red 29 |
| 815 | 33667517 | fat cholesterol | 87186 | 53 | fast scarlet ba | Biebrich scarlet |
| 816 | 33667517 | fat cholesterol | 87427 | 53 | lauric acid ethyl ester | ethyl laurate |
| 817 | 33667517 | fat cholesterol | 87430 | 53 | capric acid ethyl ester | ethyl decanoate |
| 818 | 33667517 | fat cholesterol | 87656 | 53 | hexanoate ester | hexanoate ester |
| 819 | 33667517 | fat cholesterol | 87657 | 53 | octanoate ester | octanoate ester |
| 820 | 33667517 | fat cholesterol | 87657 | 53 | caprylate ester | octanoate ester |
| 821 | 33667517 | fat cholesterol | 87658 | 53 | decanoate ester | decanoate ester |
| 822 | 33667517 | fat cholesterol | 87659 | 53 | a dodecanoate ester | dodecanoate ester |
| 823 | 33667517 | fat cholesterol | 87691 | 53 | myristate ester | tetradecanoate ester |
| 824 | 33667517 | fat cholesterol | 88753 | 53 | 22:1 ( 13z ) cholesterol ester | CE(22:1(13Z)) |
| 825 | 33667517 | fat cholesterol | 88763 | 53 | cholesteryl eicosanoate | CE(20:0) |
| 826 | 33667517 | fat cholesterol | 88768 | 53 | 18:1 ( 11z ) cholesterol ester | (11Z-octadecenoyl)-cholesterol |
| 827 | 33667517 | fat cholesterol | 88795 | 53 | 20:1 ( 11z ) cholesterol ester | CE(20:1(11Z)) |
| 828 | 33667517 | fat cholesterol | 88795 | 53 | cholesteryl eicosenoate | CE(20:1(11Z)) |
| 829 | 33667517 | fat cholesterol | 131698 | 53 | pregnane sterol | pregnane sterol |
| 830 | 33667517 | fat cholesterol | 131747 | 53 | lanostane ester | lanostane ester |
| 831 | 33667517 | fat cholesterol | 132314 | 53 | demethylzeylasteral | demethylzeylasteral |
| 832 | 33667517 | fat cholesterol | 134880 | 53 | l-dopa methyl ester | melevodopa |
| 833 | 33667517 | fat cholesterol | 135790 | 53 | succinylestriol | estriol succinate |
| 834 | 33667517 | fat cholesterol | 138115 | 53 | ltc4 , methyl ester | leukotriene C4 methyl ester |
| 835 | 33667517 | fat cholesterol | 138126 | 53 | lte4 , methyl ester | leukotriene E4 methyl ester |
| 836 | 33667517 | fat cholesterol | 141471 | 53 | gm2-methylester | ganglioside GM2 (18:0) methyl ester |
| 837 | 33667517 | fat cholesterol | 145029 | 53 | glucosilsteviol | steviolmonoside |
| 838 | 33667517 | fat cholesterol | 145213 | 53 | ( 5e ) -cholecalciferol | 5,6-trans-vitamin D3 |
| 839 | 33667517 | fat cholesterol | 146131 | 53 | 4alpha-formylzymosterol | 4alpha-formylzymosterol |
| 840 | 33667517 | fat cholesterol | 157603 | 53 | isotrichodermol | isotrichodermol |
| 841 | 33667517 | fat cholesterol | 167166 | 53 | 3-oxocholestane | 3-oxocholestane |
| 842 | 33667517 | fat cholesterol | 167166 | 53 | cholestan-3-one | 3-oxocholestane |
| 843 | 33667517 | fat cholesterol | 167708 | 53 | fty720 methyl ether | 2-amino-2-(methoxymethyl)-4-(4-octylphenyl)butan-1-ol |
| 844 | 33667517 | fat cholesterol | 174690 | 53 | ( +- ) -clenbuterol | clenbuterol |
| 845 | 33667517 | fat cholesterol | 1189 | 52 | 2-methoxyestrone | 2-methoxyestrone |
| 846 | 33667517 | fat cholesterol | 1294 | 52 | 20alpha,22beta-dihydroxycholesterol | (20R,22R)-20,22-dihydroxycholesterol |
| 847 | 33667517 | fat cholesterol | 2716 | 52 | anethole | anethole |
| 848 | 33667517 | fat cholesterol | 2904 | 52 | atenolol | atenolol |
| 849 | 33667517 | fat cholesterol | 2908 | 52 | athyriol | athyriol |
| 850 | 33667517 | fat cholesterol | 3291 | 52 | cafestol | cafestol |
| 851 | 33667517 | fat cholesterol | 3531 | 52 | centarol | Centarol |
| 852 | 33667517 | fat cholesterol | 4712 | 52 | hectorol | doxercalciferol |
| 853 | 33667517 | fat cholesterol | 4867 | 52 | estragol | Estragole |
| 854 | 33667517 | fat cholesterol | 5120 | 52 | fluoxymesteronum | fluoxymesterone |
| 855 | 33667517 | fat cholesterol | 5147 | 52 | formoterolum | formoterol |
| 856 | 33667517 | fat cholesterol | 6386 | 52 | lathyrol | Lathyrol |
| 857 | 33667517 | fat cholesterol | 6413 | 52 | letrozol | letrozole |
| 858 | 33667517 | fat cholesterol | 8082 | 52 | acetylphenol | phenyl acetate |
| 859 | 33667517 | fat cholesterol | 8379 | 52 | codelsol | prednisolone sodium phosphate |
| 860 | 33667517 | fat cholesterol | 8427 | 52 | serterol | probucol |
| 861 | 33667517 | fat cholesterol | 8629 | 52 | pterosterone | Pterosterone |
| 862 | 33667517 | fat cholesterol | 8746 | 52 | levalbuterol | (R)-salbutamol |
| 863 | 33667517 | fat cholesterol | 9401 | 52 | taraxasterol | taraxasterol |
| 864 | 33667517 | fat cholesterol | 9401 | 52 | alpha-lactucerol | taraxasterol |
| 865 | 33667517 | fat cholesterol | 9622 | 52 | tolterodinum | tolterodine |
| 866 | 33667517 | fat cholesterol | 10100 | 52 | accolate | zafirlukast |
| 867 | 33667517 | fat cholesterol | 15350 | 52 | acetyl phosphate | acetyl dihydrogen phosphate |
| 868 | 33667517 | fat cholesterol | 15362 | 52 | acetol phosphate | hydroxyacetone phosphate |
| 869 | 33667517 | fat cholesterol | 16032 | 52 | androsterone | androsterone |
| 870 | 33667517 | fat cholesterol | 16101 | 52 | aethylbenzol | ethylbenzene |
| 871 | 33667517 | fat cholesterol | 16899 | 52 | osmitrol | D-mannitol |
| 872 | 33667517 | fat cholesterol | 16966 | 52 | heteroglycan | heteroglycan |
| 873 | 33667517 | fat cholesterol | 17026 | 52 | progesterone | progesterone |
| 874 | 33667517 | fat cholesterol | 17113 | 52 | erythro-tetritol | erythritol |
| 875 | 33667517 | fat cholesterol | 17263 | 52 | oestrone | estrone |
| 876 | 33667517 | fat cholesterol | 17278 | 52 | cholestane-3,7,12,27-tetrol | 5beta-cholestane-3alpha,7alpha,12alpha,26-tetrol |
| 877 | 33667517 | fat cholesterol | 17278 | 52 | cholestane-3,7,12,26-tetrol | 5beta-cholestane-3alpha,7alpha,12alpha,26-tetrol |
| 878 | 33667517 | fat cholesterol | 17347 | 52 | testosterone | testosterone |
| 879 | 33667517 | fat cholesterol | 17347 | 52 | testosterona | testosterone |
| 880 | 33667517 | fat cholesterol | 17389 | 52 | a 2-acylglycerol | 2-monoglyceride |
| 881 | 33667517 | fat cholesterol | 17408 | 52 | acylglycerol | monoacylglycerol |
| 882 | 33667517 | fat cholesterol | 17437 | 52 | methyl thioether | dimethyl sulfide |
| 883 | 33667517 | fat cholesterol | 17456 | 52 | dithioerythritol | dithioerythritol |
| 884 | 33667517 | fat cholesterol | 18035 | 52 | a diacylglycerol | diglyceride |
| 885 | 33667517 | fat cholesterol | 18135 | 52 | catechol | catechol |
| 886 | 33667517 | fat cholesterol | 18145 | 52 | alpha-tocopherol | (R,R,R)-alpha-tocopherol |
| 887 | 33667517 | fat cholesterol | 18185 | 52 | gamma-tocopherol | gamma-tocopherol |
| 888 | 33667517 | fat cholesterol | 18431 | 52 | 7-alpha,27-dihydroxycholesterol | 7alpha,26-dihydroxycholesterol |
| 889 | 33667517 | fat cholesterol | 23051 | 52 | castasterone | castasterone |
| 890 | 33667517 | fat cholesterol | 23169 | 52 | cholate salt | cholate salt |
| 891 | 33667517 | fat cholesterol | 23382 | 52 | cu-s cluster | copper-sulfur cluster |
| 892 | 33667517 | fat cholesterol | 23783 | 52 | naphthalenediols | naphthalenediols |
| 893 | 33667517 | fat cholesterol | 23871 | 52 | palmitic acid dodecyl ester | dodecyl palmitate |
| 894 | 33667517 | fat cholesterol | 25837 | 52 | palmitoleoyl | palmitoleoyl group |
| 895 | 33667517 | fat cholesterol | 26118 | 52 | phytoecdysteroid | phytoecdysteroid |
| 896 | 33667517 | fat cholesterol | 26124 | 52 | phytosteroid | phytosteroid |
| 897 | 33667517 | fat cholesterol | 26675 | 52 | silanetetrol | silicic acid |
| 898 | 33667517 | fat cholesterol | 26753 | 52 | stearoyl | stearoyl group |
| 899 | 33667517 | fat cholesterol | 26764 | 52 | hormone steroide | steroid hormone |
| 900 | 33667517 | fat cholesterol | 26981 | 52 | threitol | threitol |
| 901 | 33667517 | fat cholesterol | 28054 | 52 | ortho-cresol | o-cresol |
| 902 | 33667517 | fat cholesterol | 28094 | 52 | trichloroethanol | 2,2,2-trichloroethanol |
| 903 | 33667517 | fat cholesterol | 28195 | 52 | aetiocholanolone | 3alpha-hydroxy-5beta-androstan-17-one |
| 904 | 33667517 | fat cholesterol | 28600 | 52 | farnesol | farnesol |
| 905 | 33667517 | fat cholesterol | 28604 | 52 | ( z ) -24-ethylidenecholesterol | isofucosterol |
| 906 | 33667517 | fat cholesterol | 28824 | 52 | stigmasterol | stigmasterol |
| 907 | 33667517 | fat cholesterol | 28865 | 52 | taurocholate | taurocholic acid |
| 908 | 33667517 | fat cholesterol | 28887 | 52 | methyl ether | dimethyl ether |
| 909 | 33667517 | fat cholesterol | 28934 | 52 | sterogyl | vitamin D2 |
| 910 | 33667517 | fat cholesterol | 29672 | 52 | acetylresorcinol | resorcinol monoacetate |
| 911 | 33667517 | fat cholesterol | 29693 | 52 | thiostrepton | thiostrepton |
| 912 | 33667517 | fat cholesterol | 29744 | 52 | lithocholate | lithocholate |
| 913 | 33667517 | fat cholesterol | 31133 | 52 | bpa catechol | 5-hydroxybisphenol A |
| 914 | 33667517 | fat cholesterol | 31398 | 52 | mycoster | ciclopirox olamine |
| 915 | 33667517 | fat cholesterol | 31409 | 52 | reactrol | Clemizole hydrochloride |
| 916 | 33667517 | fat cholesterol | 31546 | 52 | thiodrol | Epitiostanol |
| 917 | 33667517 | fat cholesterol | 31588 | 52 | fabesetronum | fabesetron |
| 918 | 33667517 | fat cholesterol | 31590 | 52 | flocalcitrol | Falecalcitriol |
| 919 | 33667517 | fat cholesterol | 31590 | 52 | flocacitriol | Falecalcitriol |
| 920 | 33667517 | fat cholesterol | 31623 | 52 | cortiplastol | fluocinolone acetonide |
| 921 | 33667517 | fat cholesterol | 31669 | 52 | exestrol | hexestrol |
| 922 | 33667517 | fat cholesterol | 31725 | 52 | amylacetic ester | isoamyl acetate |
| 923 | 33667517 | fat cholesterol | 32232 | 52 | gastirol | tiquizium bromide |
| 924 | 33667517 | fat cholesterol | 32372 | 52 | palmitoleate | palmitoleate |
| 925 | 33667517 | fat cholesterol | 33111 | 52 | apholate | apholate |
| 926 | 33667517 | fat cholesterol | 33235 | 52 | tocotrienols | tocotrienol |
| 927 | 33667517 | fat cholesterol | 33270 | 52 | zeta1-tocopherol | alpha-tocotrienol |
| 928 | 33667517 | fat cholesterol | 33275 | 52 | beta-tocotrienol | beta-tocotrienol |
| 929 | 33667517 | fat cholesterol | 33308 | 52 | carboxylic ester | carboxylic ester |
| 930 | 33667517 | fat cholesterol | 33729 | 52 | tetritol | tetritol |
| 931 | 33667517 | fat cholesterol | 33838 | 52 | a nucleoside | nucleoside |
| 932 | 33667517 | fat cholesterol | 33853 | 52 | a phenol | phenols |
| 933 | 33667517 | fat cholesterol | 34165 | 52 | 16-ketoestradiol | 16-Ketoestradiol |
| 934 | 33667517 | fat cholesterol | 34227 | 52 | archaeol | 2,3-di-O-phytanyl-sn-glycerol |
| 935 | 33667517 | fat cholesterol | 34749 | 52 | ethisteronum | ethisterone |
| 936 | 33667517 | fat cholesterol | 34829 | 52 | hydroxymesterone | Medrysone |
| 937 | 33667517 | fat cholesterol | 34903 | 52 | oxymesterone | Oranabol |
| 938 | 33667517 | fat cholesterol | 35047 | 52 | xanthomicrol | xanthomicrol |
| 939 | 33667517 | fat cholesterol | 35287 | 52 | acylcholines | acylcholine |
| 940 | 33667517 | fat cholesterol | 35348 | 52 | 3beta-sterol | 3beta-sterol |
| 941 | 33667517 | fat cholesterol | 35517 | 52 | pseudocholestane | 5beta-cholestane |
| 942 | 33667517 | fat cholesterol | 35682 | 52 | naphthalenol | naphthol |
| 943 | 33667517 | fat cholesterol | 35759 | 52 | a 1-acylglycerol | 1-monoglyceride |
| 944 | 33667517 | fat cholesterol | 35788 | 52 | seco-steroid | seco-steroid |
| 945 | 33667517 | fat cholesterol | 35789 | 52 | keto steroid | oxo steroid |
| 946 | 33667517 | fat cholesterol | 35915 | 52 | steryl ester | sterol ester |
| 947 | 33667517 | fat cholesterol | 36243 | 52 | propanoate ester | propanoate ester |
| 948 | 33667517 | fat cholesterol | 36598 | 52 | heterone | heterone |
| 949 | 33667517 | fat cholesterol | 36818 | 52 | seco-cholestanes | seco-cholestane |
| 950 | 33667517 | fat cholesterol | 37505 | 52 | anthracenetriols | anthracenetriol |
| 951 | 33667517 | fat cholesterol | 37864 | 52 | thioperoxols | thioperoxol |
| 952 | 33667517 | fat cholesterol | 38138 | 52 | naphthylmethanol | naphthylmethanol |
| 953 | 33667517 | fat cholesterol | 40968 | 52 | ovoester | astaxanthin |
| 954 | 33667517 | fat cholesterol | 41633 | 52 | ( 24s ) -24,25-epoxycholesterol | 24(S),25-epoxycholesterol |
| 955 | 33667517 | fat cholesterol | 42090 | 52 | l-threo-tetritol | L-threitol |
| 956 | 33667517 | fat cholesterol | 42471 | 52 | coleonol | forskolin |
| 957 | 33667517 | fat cholesterol | 45619 | 52 | d-1-thioglycerol | (R)-monothioglycerol |
| 958 | 33667517 | fat cholesterol | 46511 | 52 | aethanethiol | ethanethiol |
| 959 | 33667517 | fat cholesterol | 46668 | 52 | amino acid ester | amino acid ester |
| 960 | 33667517 | fat cholesterol | 47772 | 52 | delta-tocopherol | delta-tocopherol |
| 961 | 33667517 | fat cholesterol | 47774 | 52 | low-density lipoprotein cholesterol | low-density lipoprotein cholesterol |
| 962 | 33667517 | fat cholesterol | 47787 | 52 | an 11-oxosteroid | 11-oxo steroid |
| 963 | 33667517 | fat cholesterol | 47836 | 52 | oxo seco-steroid | oxo seco-steroid |
| 964 | 33667517 | fat cholesterol | 48056 | 52 | acetonyl | acetonyl group |
| 965 | 33667517 | fat cholesterol | 48300 | 52 | d-threo-tetritol | D-threitol |
| 966 | 33667517 | fat cholesterol | 48873 | 52 | anticholinergika | cholinergic antagonist |
| 967 | 33667517 | fat cholesterol | 48873 | 52 | anticolinergicos | cholinergic antagonist |
| 968 | 33667517 | fat cholesterol | 50477 | 52 | n-butyrate ester | butyrate ester |
| 969 | 33667517 | fat cholesterol | 50539 | 52 | thiolate | thiolate anion |
| 970 | 33667517 | fat cholesterol | 50646 | 52 | antiosteoporotic | bone density conservation agent |
| 971 | 33667517 | fat cholesterol | 50749 | 52 | calcipotriol | calcipotriol |
| 972 | 33667517 | fat cholesterol | 50751 | 52 | antiestrogen | anti-estrogen |
| 973 | 33667517 | fat cholesterol | 50777 | 52 | etonogestrel | etonogestrel |
| 974 | 33667517 | fat cholesterol | 50786 | 52 | anabolic steriod | anabolic androgenic steroid |
| 975 | 33667517 | fat cholesterol | 50858 | 52 | corticosteroides | corticosteroid |
| 976 | 33667517 | fat cholesterol | 50871 | 52 | pentanoate ester | valerate ester |
| 977 | 33667517 | fat cholesterol | 50898 | 52 | heptanoate ester | heptanoate ester |
| 978 | 33667517 | fat cholesterol | 51211 | 52 | alfeprol | alprenolol |
| 979 | 33667517 | fat cholesterol | 51848 | 52 | alpha-keto-ester | alpha-ketoester |
| 980 | 33667517 | fat cholesterol | 51848 | 52 | alpha-keto ester | alpha-ketoester |
| 981 | 33667517 | fat cholesterol | 51848 | 52 | alpha-ketoesters | alpha-ketoester |
| 982 | 33667517 | fat cholesterol | 52320 | 52 | ergosterol ester | ergosteryl ester |
| 983 | 33667517 | fat cholesterol | 52322 | 52 | zymosterol ester | zymosterol ester |
| 984 | 33667517 | fat cholesterol | 52343 | 52 | formic ester | formate ester |
| 985 | 33667517 | fat cholesterol | 52394 | 52 | lanosteryl ester | lanosteryl ester |
| 986 | 33667517 | fat cholesterol | 53274 | 52 | glyoxylate ester | glyoxylate ester |
| 987 | 33667517 | fat cholesterol | 53519 | 52 | graft co-polymer | graft copolymer |
| 988 | 33667517 | fat cholesterol | 59722 | 52 | thioenol | thioenol |
| 989 | 33667517 | fat cholesterol | 60297 | 52 | cmo-testosterone | testosterone 3-(O-carboxymethyl)oxime |
| 990 | 33667517 | fat cholesterol | 60528 | 52 | lysergol | lysergol |
| 991 | 33667517 | fat cholesterol | 60660 | 52 | 5-chlorovaleroyl | 5-chloropentanoyl group |
| 992 | 33667517 | fat cholesterol | 60782 | 52 | fluprostenol | fluprostenol |
| 993 | 33667517 | fat cholesterol | 61655 | 52 | glycosteroid | steroid saponin |
| 994 | 33667517 | fat cholesterol | 61655 | 52 | glycosyl steroid | steroid saponin |
| 995 | 33667517 | fat cholesterol | 62183 | 52 | anthrose | beta-anthropyranose |
| 996 | 33667517 | fat cholesterol | 62513 | 52 | machilol | gamma-eudesmol |
| 997 | 33667517 | fat cholesterol | 64729 | 52 | glutathioselenol | glutathioselenol |
| 998 | 33667517 | fat cholesterol | 65858 | 52 | peroxyergosterol | ergosterol peroxide |
| 999 | 33667517 | fat cholesterol | 65888 | 52 | eurysterol a | eurysterol A |
| 1000 | 33667517 | fat cholesterol | 65889 | 52 | eurysterol b | eurysterol B |
| 1001 | 33667517 | fat cholesterol | 66960 | 52 | olivetol | olivetol |
| 1002 | 33667517 | fat cholesterol | 67237 | 52 | cholest-5-en-3beta,22r-diol | (22R)-22-hydroxycholesterol |
| 1003 | 33667517 | fat cholesterol | 67554 | 52 | sterolin | daucosterol |
| 1004 | 33667517 | fat cholesterol | 67897 | 52 | farrerol | farrerol |
| 1005 | 33667517 | fat cholesterol | 68257 | 52 | platensic acid methyl ester | platensic acid methyl ester |
| 1006 | 33667517 | fat cholesterol | 69620 | 52 | trichothecinol b | Trichothecinol B |
| 1007 | 33667517 | fat cholesterol | 70316 | 52 | ergosterol d | Ergosterol D |
| 1008 | 33667517 | fat cholesterol | 73420 | 52 | fty720 phosphate | fingolimod phosphate |
| 1009 | 33667517 | fat cholesterol | 74537 | 52 | monothioglycerol | monothioglycerol |
| 1010 | 33667517 | fat cholesterol | 75628 | 52 | arachidyl oleate | arachidyl oleate |
| 1011 | 33667517 | fat cholesterol | 76003 | 52 | afatinib maleate | afatinib dimaleate |
| 1012 | 33667517 | fat cholesterol | 76943 | 52 | anti-copalol | (+)-copalol |
| 1013 | 33667517 | fat cholesterol | 76990 | 52 | mycoestrogen | mycoestrogen |
| 1014 | 33667517 | fat cholesterol | 78801 | 52 | oxathiolanes | oxathiolane |
| 1015 | 33667517 | fat cholesterol | 79512 | 52 | 4-nitroestradiol | 4-Nitroestra-1,3,5(10)-triene-3,17beta-diol |
| 1016 | 33667517 | fat cholesterol | 79529 | 52 | 2-aminoestradiol | 2-Aminoestra-1,3,5(10)-triene-3,17beta-diol |
| 1017 | 33667517 | fat cholesterol | 79530 | 52 | 2-nitroestradiol | 2-Nitroestra-1,3,5(10)-triene-3,17beta-diol |
| 1018 | 33667517 | fat cholesterol | 80929 | 52 | costatol | Costatol |
| 1019 | 33667517 | fat cholesterol | 81485 | 52 | osthenol | osthenol |
| 1020 | 33667517 | fat cholesterol | 81766 | 52 | chlortriafol | hexaconazole |
| 1021 | 33667517 | fat cholesterol | 81781 | 52 | ethyltrianol | tebuconazole |
| 1022 | 33667517 | fat cholesterol | 81990 | 52 | ethofumesate | ethofumesate |
| 1023 | 33667517 | fat cholesterol | 82405 | 52 | acetoxyl | Benzoyl peroxide |
| 1024 | 33667517 | fat cholesterol | 82946 | 52 | butyl oleate | n-butyl oleate |
| 1025 | 33667517 | fat cholesterol | 83455 | 52 | tolutriazole | 5-methyl-1H-benzotriazole |
| 1026 | 33667517 | fat cholesterol | 83703 | 52 | thioglycerolipid | monothioglycerolipid |
| 1027 | 33667517 | fat cholesterol | 84940 | 52 | ethyl oleate | ethyl oleate |
| 1028 | 33667517 | fat cholesterol | 86074 | 52 | 24s,25-dihydroxycholesterol | (24S,25)-dihydroxycholesterol |
| 1029 | 33667517 | fat cholesterol | 86075 | 52 | 24s,27-dihydroxycholesterol | (24S,26)-dihydroxycholesterol |
| 1030 | 33667517 | fat cholesterol | 86145 | 52 | 7alpha,22r-dihydroxycholesterol | (7alpha,22R)-dihydroxycholesterol |
| 1031 | 33667517 | fat cholesterol | 87627 | 52 | fast blue salt b | fast blue salt B |
| 1032 | 33667517 | fat cholesterol | 88156 | 52 | diacetylglycerol | diacetin |
| 1033 | 33667517 | fat cholesterol | 88258 | 52 | sulfametrole | sulfametrole |
| 1034 | 33667517 | fat cholesterol | 88758 | 52 | cholesteryl 1-myristoleoate | CE(14:1(9Z)) |
| 1035 | 33667517 | fat cholesterol | 88758 | 52 | cholesterol 1-myristoleoate | CE(14:1(9Z)) |
| 1036 | 33667517 | fat cholesterol | 89029 | 52 | decanoic acid cholesteryl ester | CE(10:0) |
| 1037 | 33667517 | fat cholesterol | 89029 | 52 | cholesterol 3-decanoic acid | CE(10:0) |
| 1038 | 33667517 | fat cholesterol | 89234 | 52 | d5-cholenate | 3b-Hydroxy-5-cholenoic acid |
| 1039 | 33667517 | fat cholesterol | 89519 | 52 | ( 3beta,5beta ) -cholestan-3-ol | coprostanol |
| 1040 | 33667517 | fat cholesterol | 89760 | 52 | solerole | Solerol |
| 1041 | 33667517 | fat cholesterol | 131642 | 52 | ergostane sterol | ergostane sterol |
| 1042 | 33667517 | fat cholesterol | 131737 | 52 | androstane ester | androstane ester |
| 1043 | 33667517 | fat cholesterol | 132340 | 52 | dihydrocelastrol | triptohypol C |
| 1044 | 33667517 | fat cholesterol | 134311 | 52 | altritol | D-altritol |
| 1045 | 33667517 | fat cholesterol | 134925 | 52 | tolseram | mephenesin carbamate |
| 1046 | 33667517 | fat cholesterol | 134934 | 52 | actebral | cyprodenate |
| 1047 | 33667517 | fat cholesterol | 135018 | 52 | metesculetol | metesculetol |
| 1048 | 33667517 | fat cholesterol | 135042 | 52 | thenitrazole | tenonitrozole |
| 1049 | 33667517 | fat cholesterol | 135175 | 52 | thoragol | bibenzonium |
| 1050 | 33667517 | fat cholesterol | 135235 | 52 | tiaterol | tiadenol |
| 1051 | 33667517 | fat cholesterol | 135264 | 52 | chemestrogen | benzestrol |
| 1052 | 33667517 | fat cholesterol | 135271 | 52 | norgesterone | norgesterone |
| 1053 | 33667517 | fat cholesterol | 135535 | 52 | tricerol | etofibrate |
| 1054 | 33667517 | fat cholesterol | 135572 | 52 | dacorsol | prednylidene |
| 1055 | 33667517 | fat cholesterol | 135716 | 52 | ceftezol | ceftezole |
| 1056 | 33667517 | fat cholesterol | 135920 | 52 | fesoterodine | fesoterodine |
| 1057 | 33667517 | fat cholesterol | 136972 | 52 | 4-methoxyestrone | 4-methoxyestrone |
| 1058 | 33667517 | fat cholesterol | 139292 | 52 | a chlorophyllide | chlorophyllide(2-) |
| 1059 | 33667517 | fat cholesterol | 142077 | 52 | abediterolum | abediterol |
| 1060 | 33667517 | fat cholesterol | 143703 | 52 | thiogeraniol | thiogeraniol |
| 1061 | 33667517 | fat cholesterol | 145763 | 52 | 6-oxoestriol | 6-ketoestriol |
| 1062 | 33667517 | fat cholesterol | 145952 | 52 | 4-formyl steroid | 4-formyl steroid |
| 1063 | 33667517 | fat cholesterol | 167207 | 52 | sterinol | benzododecinium bromide |
| 1064 | 33667517 | fat cholesterol | 167379 | 52 | trichobrasilenol | trichobrasilenol |
| 1065 | 33667517 | fat cholesterol | 172390 | 52 | glycol ether | glycol ether |
| 1066 | 33667517 | fat cholesterol | 178024 | 52 | 26-oxolanosterol | 26-oxolanosterol |
| 1067 | 33667517 | fat cholesterol | 408174 | 52 | arformoterol | arformoterol |
| 1068 | 33667517 | fat cholesterol | 1294 | 51 | 20alpha,22r-dihydroxycholesterol | (20R,22R)-20,22-dihydroxycholesterol |
| 1069 | 33667517 | fat cholesterol | 4591 | 51 | dihydrotachysterolum | dihydrotachysterol |
| 1070 | 33667517 | fat cholesterol | 4893 | 51 | acetoacetic acid ethyl ester | ethyl acetoacetate |
| 1071 | 33667517 | fat cholesterol | 4903 | 51 | 17alpha-ethynylestradiol | 17alpha-ethynylestradiol |
| 1072 | 33667517 | fat cholesterol | 7794 | 51 | phosphate monoesters | phosphate monoester |
| 1073 | 33667517 | fat cholesterol | 16608 | 51 | 5alpha-cholest-8-en-3beta-ol | 5alpha-cholest-8-en-3beta-ol |
| 1074 | 33667517 | fat cholesterol | 17026 | 51 | 17alpha-progesterone | progesterone |
| 1075 | 33667517 | fat cholesterol | 17168 | 51 | 5alpha-cholest-7-en-3beta-ol | 5alpha-cholest-7-en-3beta-ol |
| 1076 | 33667517 | fat cholesterol | 22327 | 51 | aliphatic thioethers | aliphatic sulfide |
| 1077 | 33667517 | fat cholesterol | 23203 | 51 | cholestenoyl-coenzyme as | cholestenoyl-CoA |
| 1078 | 33667517 | fat cholesterol | 26959 | 51 | thiocarboxylic ester | thiocarboxylic ester |
| 1079 | 33667517 | fat cholesterol | 27910 | 51 | 7-dehydrodesmosterol | 7-dehydrodesmosterol |
| 1080 | 33667517 | fat cholesterol | 28575 | 51 | l-glutamate methyl ester | L-glutamate methyl ester |
| 1081 | 33667517 | fat cholesterol | 34142 | 51 | 11beta-chloromethylestradiol | 11beta-Chloromethylestradiol |
| 1082 | 33667517 | fat cholesterol | 34400 | 51 | 4-chloroprogesterone | 4-Chloroprogesterone |
| 1083 | 33667517 | fat cholesterol | 34896 | 51 | methylnortestosteron | Normethandrolone |
| 1084 | 33667517 | fat cholesterol | 35865 | 51 | 5alpha-cholest-7-ene | 5alpha-cholest-7-ene |
| 1085 | 33667517 | fat cholesterol | 37733 | 51 | cholinesterase inhibitor | EC 3.1.1.8 (cholinesterase) inhibitor |
| 1086 | 33667517 | fat cholesterol | 38094 | 51 | arenesulfonate ester | arenesulfonate ester |
| 1087 | 33667517 | fat cholesterol | 38462 | 51 | acetylcholine esterase inhibitor | EC 3.1.1.7 (acetylcholinesterase) inhibitor |
| 1088 | 33667517 | fat cholesterol | 41321 | 51 | cholesterol hydrogen sulfate | cholesterol sulfate |
| 1089 | 33667517 | fat cholesterol | 41922 | 51 | trans-diethylstilbestrol | diethylstilbestrol |
| 1090 | 33667517 | fat cholesterol | 41922 | 51 | diethylstilbestrolum | diethylstilbestrol |
| 1091 | 33667517 | fat cholesterol | 47775 | 51 | high-density lipoprotein cholesterol | high-density lipoprotein cholesterol |
| 1092 | 33667517 | fat cholesterol | 50516 | 51 | cholest-5-ene-3beta,24r-diol | (24R)-24-hydroxycholesterol |
| 1093 | 33667517 | fat cholesterol | 50569 | 51 | acetic acid , isobutyl ester | isobutyl acetate |
| 1094 | 33667517 | fat cholesterol | 57589 | 51 | l-carnitine acetyl ester | O-acetyl-L-carnitine |
| 1095 | 33667517 | fat cholesterol | 59047 | 51 | nonoate methyl ester lewis y | methyl 8-{[alpha-L-fucopyranosyl-(1->3)-[alpha-L-fucopyranosyl-(1->2)-beta-D-galactopyranosyl-(1->4)]-2-acetamido-2-deoxy-beta-D-glucopyranosyl]oxy}nonanoate |
| 1096 | 33667517 | fat cholesterol | 64683 | 51 | a 1-acyl-sn-glycerol | 1-acyl-sn-glycerol |
| 1097 | 33667517 | fat cholesterol | 65331 | 51 | alkyl caffeate ester | alkyl caffeate ester |
| 1098 | 33667517 | fat cholesterol | 68505 | 51 | pyrocatechol sulfate | pyrocatechol sulfate |
| 1099 | 33667517 | fat cholesterol | 68505 | 51 | catechol monosulfate | pyrocatechol sulfate |
| 1100 | 33667517 | fat cholesterol | 69656 | 51 | caffeic acid ethyl ester | Caffeic acid ethyl ester |
| 1101 | 33667517 | fat cholesterol | 72999 | 51 | palmitate de colfosceril | 1,2-dihexadecanoyl-sn-glycero-3-phosphocholine |
| 1102 | 33667517 | fat cholesterol | 73224 | 51 | volatile mustard oil | allyl isothiocyanate |
| 1103 | 33667517 | fat cholesterol | 75620 | 51 | decanol oleate ester | decyl oleate |
| 1104 | 33667517 | fat cholesterol | 77006 | 51 | benzoate d'estradiol | 17beta-estradiol 3-benzoate |
| 1105 | 33667517 | fat cholesterol | 77086 | 51 | l-carnitine dodecanoyl ester | O-lauroyl-L-carnitine |
| 1106 | 33667517 | fat cholesterol | 77699 | 51 | formic acid methyl ester | methyl formate |
| 1107 | 33667517 | fat cholesterol | 77845 | 51 | 7-aminocholest-5-en-3beta-ol | 7-aminocholesterol |
| 1108 | 33667517 | fat cholesterol | 79417 | 51 | 6beta-methyltestosterone | 17beta-Hydroxy-6beta-methylandrost-4-en-3-one |
| 1109 | 33667517 | fat cholesterol | 79913 | 51 | 4-methyltestosterone | 17beta-Hydroxy-4-methylandrost-4-en-3-one |
| 1110 | 33667517 | fat cholesterol | 80097 | 51 | 5-dehydroavenasterol | 5-Dehydroavenasterol |
| 1111 | 33667517 | fat cholesterol | 82751 | 51 | cholesteryl arachidonate | cholesteryl arachidonate |
| 1112 | 33667517 | fat cholesterol | 83022 | 51 | glucosyltestosterone | glucosyltestosterone |
| 1113 | 33667517 | fat cholesterol | 83344 | 51 | 8-hpete methyl ester | 8-HPETE methyl ester |
| 1114 | 33667517 | fat cholesterol | 83621 | 51 | fatty acyl-adenylate | fatty acyl-AMP |
| 1115 | 33667517 | fat cholesterol | 83746 | 51 | 3-ketocholest-4-en-26-al | 3-ketocholest-4-en-26-al |
| 1116 | 33667517 | fat cholesterol | 84934 | 51 | palmitoleic acid ethyl ester | ethyl palmitoleate |
| 1117 | 33667517 | fat cholesterol | 84936 | 51 | stearic acid ethyl ester | ethyl octadecanoate |
| 1118 | 33667517 | fat cholesterol | 86023 | 51 | alpha-eleostearoyl group | (9Z,11E,13E)-octadecatrienoyl group |
| 1119 | 33667517 | fat cholesterol | 86055 | 51 | caproic acid ethyl ester | ethyl hexanoate |
| 1120 | 33667517 | fat cholesterol | 86074 | 51 | ( 24s,25 ) -dihydroxycholesterol | (24S,25)-dihydroxycholesterol |
| 1121 | 33667517 | fat cholesterol | 86075 | 51 | ( 24s,26 ) -dihydroxycholesterol | (24S,26)-dihydroxycholesterol |
| 1122 | 33667517 | fat cholesterol | 86145 | 51 | ( 7alpha,22r ) -dihydroxycholesterol | (7alpha,22R)-dihydroxycholesterol |
| 1123 | 33667517 | fat cholesterol | 86145 | 51 | ( 22r,7alpha ) -dihydroxycholesterol | (7alpha,22R)-dihydroxycholesterol |
| 1124 | 33667517 | fat cholesterol | 87494 | 51 | lauric acid methyl ester | methyl laurate |
| 1125 | 33667517 | fat cholesterol | 88740 | 51 | cholesterol 1-tetracosanoate | CE(24:0) |
| 1126 | 33667517 | fat cholesterol | 88752 | 51 | cholesterol ester ( 20:3n9 ) | cholesteryl (5Z,8Z,11Z)-icosatrienoate |
| 1127 | 33667517 | fat cholesterol | 88754 | 51 | cholesteryl 1-osbondoate | CE(22:5(4Z,7Z,10Z,13Z,16Z)) |
| 1128 | 33667517 | fat cholesterol | 88754 | 51 | cholesterol 1-osbondoic acid | CE(22:5(4Z,7Z,10Z,13Z,16Z)) |
| 1129 | 33667517 | fat cholesterol | 88756 | 51 | cholesteryl gamma-linolenate | cholesteryl gamma-linolenate |
| 1130 | 33667517 | fat cholesterol | 88763 | 51 | eicosanoic acidcholesteryl ester | CE(20:0) |
| 1131 | 33667517 | fat cholesterol | 88764 | 51 | butyric acid ethyl ester | ethyl butyrate |
| 1132 | 33667517 | fat cholesterol | 131420 | 51 | ethylcholine mustard | 2-chloro-2'-hydroxytriethylamine |
| 1133 | 33667517 | fat cholesterol | 133940 | 51 | lithocholate sulfate | lithocholate sulfate(2-) |
| 1134 | 33667517 | fat cholesterol | 135050 | 51 | aspirin phenyl ester | phenyl acetylsalicylate |
| 1135 | 33667517 | fat cholesterol | 135336 | 51 | ibuprofen guaiacol ester | ibuprofen guaiacol ester |
| 1136 | 33667517 | fat cholesterol | 135971 | 51 | levodopa ethyl ester | etilevodopa |
| 1137 | 33667517 | fat cholesterol | 136838 | 51 | 2-methoxyethyl ester | 2-methoxyethyl ester |
| 1138 | 33667517 | fat cholesterol | 138665 | 51 | malic acid 4-ethyl ester | 4-ethoxy-2-hydroxy-4-oxobutanoic acid |
| 1139 | 33667517 | fat cholesterol | 141525 | 51 | ochratoxin a ethyl ester | ochratoxin C |
| 1140 | 33667517 | fat cholesterol | 141701 | 51 | catechol monoethyl ether | 2-ethoxyphenol |
| 1141 | 33667517 | fat cholesterol | 142495 | 51 | 1-arachidoylglycerol | 1-icosanoylglycerol |
| 1142 | 33667517 | fat cholesterol | 145032 | 51 | 20-hete methyl ester | 20-HETE methyl ester |
| 1143 | 33667517 | fat cholesterol | 145828 | 51 | gallic acid methyl ester | methyl 3,4,5-trihydroxybenzoate |
| 1144 | 33667517 | fat cholesterol | 145969 | 51 | 5-tricosylresorcinol | 5-tricosylresorcinol |
| 1145 | 33667517 | fat cholesterol | 155845 | 51 | cholest-4-en-25-ol-3-one | 25-hydroxycholest-4-en-3-one |
| 1146 | 33667517 | fat cholesterol | 166888 | 51 | 7-dehydroavenasterol | avenasterol |
| 1147 | 33667517 | fat cholesterol | 176902 | 51 | a 14alpha-formyl steroid | 14alpha-formyl steroid |
| 1148 | 33667517 | fat cholesterol | 177027 | 51 | dodecanoate phytyl ester | laurate phytyl ester |
| 1149 | 33667517 | fat cholesterol | 178023 | 51 | 26-hydroxylanosterol | 26-hydroxylanosterol |
